# Chitosan siRNA Nanoparticles Produce Significant Non-Toxic Functional Gene Silencing in Kidney Cortices

**DOI:** 10.3390/polym16172547

**Published:** 2024-09-09

**Authors:** Mohamad-Gabriel Alameh, Ashkan Tavakoli Naeini, Garima Dwivedi, Frederic Lesage, Michael D. Buschmann, Marc Lavertu

**Affiliations:** 1 Institute of Biomedical Engineering, Polytechnique Montreal, 2500 Chem. de Polytechnique, Montréal, QC H3T 1J4, Canada; tavakoli-naeini.ashkan@polymtl.ca (A.T.N.); garima.dwivedi@pennmedicine.upenn.edu (G.D.); frederic.lesage@polymtl.ca (F.L.);; 2 Department of Chemical Engineering, Polytechnique Montreal, 2500 Chem. de Polytechnique, Montreal, QC H3T 1J4, Canada

**Keywords:** chitosan, toxicity, Invivofectamine, cationic lipid nanoparticles, siRNA, hyaluronic acid

## Abstract

Chitosan shows effective nucleic acid delivery. To understand the influence of chitosan’s molecular weight, dose, payload, and hyaluronic acid coating on in vivo toxicity, immune stimulation, biodistribution and efficacy, precisely characterized chitosans were formulated with unmodified or chemically modified siRNA to control for innate immune stimulation. The hemocompatibility, cytokine induction, hematological and serological responses were assessed. Body weight, clinical signs, in vivo biodistribution and functional target knockdown were monitored. Hemolysis was found to be dose- and MW-dependent with the HA coating abrogating hemolysis. Compared to cationic lipid nanoparticles, uncoated and HA-coated chitosan nanoparticles did not induce immune stimulation or hematologic toxicity. Liver and kidney biomarkers remained unchanged with chitosan formulations, while high doses of cationic lipid nanoparticles led to increased transaminase levels and a decrease in body weight. Uncoated and HA-coated nanoparticles accumulated in kidneys with functional knockdown for uncoated chitosan formulations reaching 60%, suggesting potential applications in the treatment of kidney diseases.

## 1. Introduction

Small interfering RNA (siRNA) shows immense potential in treating previously un-druggable diseases via gene-specific knockdown as demonstrated by the FDA approval of ONPATTRO^®^ (partisiran) [1] and GIVLAARI^®^ (givosiran) [2]. While the chemical modification of siRNA increased the nuclease resistance, serum stability, target affinity and half-life of the molecule, encapsulation into lipid nanoparticles (LNPs) or conjugation with targeting ligands (e.g., N-acetylgalactosamine) improved the therapeutic window and altered the distribution and pharmacokinetics profiles [3]. LNPs and GalNac siRNA conjugates are mostly restricted to the liver following intravenous (I.V.) administration and require steroidal anti-inflammatory treatment preceding LNP dosing to limit cytokine production. Delivery systems that meet criteria such as colloidal stability, high encapsulation efficiency, low toxicity/immune stimulation, and siRNA delivery efficiency to extrahepatic organs are critically needed.

Chitosan (CS), a family of cationic bio-copolymers composed of β (1-4) linked N-acetyl glucosamine (GlcNAc) and D-glucosamine (Glc), gained attention for nucleic acid (NA) delivery due to its low toxicity, simple production, and ease of chemical modification [3,4]. It can be tweaked for specific fractions of protonable Glc vs. GlcNAc, average molecular weights (Mw and Mn), and assembly into polyelectrolyte complexes (nanoparticles) via spontaneous electrostatic interactions. Several in vitro studies showing siRNA delivery with chitosan have been published previously [4,5,6,7,8,9,10,11,12,13,14,15,16,17,18,19,20,21,22,23,24,25]. In vivo, the nasal administration and intratracheal catheter administration of chitosan/siRNA formulations led to effective RNA interference in the lungs of transgenic EGFP mice [15,21]. A sustained and effective siRNA accumulation of chitosan/siRNA formulation was shown within the kidneys of I.V. injected mice [26,27]. Folic acid/chitosan conjugates were used to deliver siRNA to activated macrophages [28]. Chitosan/siRNA nanoparticles were shown to knock down COX-2 specifically in macrophages, which might prevent kidney injury induced by unilateral ureteral obstruction [29]. Since the in vivo induction of cytokines was never extensively characterized in previous work, a systemic study with accurately characterized chitosans that investigates hemocompatibility, in vivo acute toxicity and demonstrates knockdown following the I.V. administration of nanoparticles (NPs) is needed.

Here, we investigated the effect of chitosan polymer length, dose, and surface modification with hyaluronic acid (HA) on the hemolytic potential, acute and organ toxicity, cytokine induction, in vivo biodistribution and target knockdown efficacy, compared chitosan NPs with commercially available cationic LNPs (Invivofectamine^®^) for siRNA delivery, and extensively assessed acute toxicity, biodistribution in live animal imaging, and target knockdown efficacy in mice. We hypothesized that the administration of sub-hemolytic doses of chitosan is non-toxic compared to LNPs and induces potent gene knockdown at the site of accumulation. We tested three specific hypotheses in this study: (1) chitosan nanoparticles accumulate extra hepatically, (2) HA-coated nanoparticles have a different biodistribution pattern vs. uncoated formulations, and (3) HA nanoparticles demonstrate higher knockdown efficiency in accumulated sites due to improved hemocompatibility and increased doses.

## 2. Materials and Methods

### 2.1. Materials

Medical grade hyaluronic acid (HA, 866 kDa, HA1M-1) was purchased from Life Core Biomedical (Life Core Biomedical LLC, Chaska, MN, USA). This particular HA was chosen based on our previous work on nanoparticle stability [30]. A lipopolysaccharide (LPS) serotype O55:B5 (TLRgrade™) from Enzo Life Sciences (Enzo life sciences, Farmingdale, NY, USA), isoflurane (Forane™) from Baxter (Baxter Canada, Mississauga, ON, Canada), BD vacutainer SST Gold from VWR international (VWR International, Mont-Royal, QC, Canada), IDEXX green top Lithium–Heparin and yellow top serum microtainers from IDEXX Laboratories (IDEXX Laboratories, Markham, ON, Canada), Altogen in vivo transfection kit from Altogen Biosystems (Altogen Biosystems, Las Vegas, NV, USA), Invivofectamine^®^ 2.0 and 3.0, phosphate-buffered saline (PBS), UltraPure™ DNase⁄RNase-Free water, 10% Neutral Buffer Formalin, AlexaFluor 546 phalloidin with ProLong^®^ Diamond antifade containing DAPI and nuclease free water from Life technologies (Burlington, ON, Canada) were all used. D-trehalose, L-histidine, diethyl pyrocarbonate (DEPC), 1N HCl, and RNaseZAP™ were purchased from Sigma-Aldrich (Sigma-Aldrich, Oakville, ON, Canada). Rabbit monoclonal anti-GAPDH (Ab181602) and biotinylated goat anti-Rabbit IgG (Ab97049) were purchased from Abcam (Abcam, Cambridge, UK). Serum vials (223685, 223686 and 223687) were purchased from Wheaton (Wheaton, Millville, NJ, USA), and butyl stoppers (73828A-21) were purchased from Kimble Chase (Kimble Chase, Rockwood, TN, USA). PVDF filters (0.22 μm) and Amicon Ultra-15 centrifugal filter units were purchased from EDM Millipore (EDM Millipore Ltd., Etobicoke, ON, Canada). The native and 2′O methyl (2′OMe) modified anti-ApoB siRNA sequences were custom synthesized by Dharmacon Inc (GE Dharmacon, Lafayette, CO, USA). The anti-GAPDH siRNA was purchased from Life technologies as a predesigned Ambion^®^ In Vivo GAPDH Positive Control siRNA (Life technologies, Burlington, ON, Canada). Chitosans were obtained from Marinard, (Laval, QC, Canada).

### 2.2. siRNA Sequences and Chitosan Characterization

All siRNA sequences came in a lyophilized format following HPLC purification and subjected to quality control (QC) (e.g., endotoxin content, LC-MS, PAGE and UV/Vis spectrophotometric analysis). The sequences of siRNAs are summarized in Table S3.

Chitosans were depolymerized with nitrous acid with the aim of obtaining chitosans of number-average Mn of 10 and 120 kDa. Those target Mn were chosen based on our previous work using chitosan to deliver siRNA [31]. The actual chitosan number and weight-average molecular weights (Mn and Mw) (Table 1) were then determined by gel permeation chromatography (GPC) using a Shimadzu LC-20AD isocratic pump coupled with a Dawn HELEOS II multi angle laser light scattering detector (Wyatt Technology Co., Santa Barbara, CA, USA), an Optilab rEX interferometric refractometer (Wyatt Technology Co.), and two Tosoh TSKgel (G6000PWxl-CP and G5000PWxl-CP; Tosoh Bioscience LLC, King of Prussia, PA, USA) columns. Chitosans were eluted at pH 4.5 using an acetic acid (0.15 M)/sodium acetate (0.1 M)/sodium azide (4 mM) buffer. The injection volume was 100 μL at an 0.8 mL/min flow rate at 25 °C. The dn/dc value was determined as 0.208 at 658 nm. The degree of deacetylation (DDA) was determined by 1H NMR.

$$Dp=\frac{Mn\;chitosan}{Average\;monomer\;molar\;mass\;at\;specific\;DDA}$$

### 2.3. Preparation of Chitosan-Based Nanoparticles

Low (10 kDa) and high (120 kDa) molecular weight chitosans were dissolved overnight in nuclease-free water (NFW) and 1N HCl, using a glucosamine to HCl ratio of 1:1, to a final concentration of 5 mg/mL. HA was prepared by dissolving sodium hyaluronate in NFW at a concentration of 1 mg/mL. The stock solutions were sterile filtered using a 0.22 μm PVDF filter and used to prepare solutions containing 0.83% *w*/*v* trehalose and 5.83 mM histidine (toxicity) or 1% trehalose and 3.8 mM histidine (efficacy) at a specific amine: phosphate: HA carboxyl molar ratio (N:P:C = 2:1:1.5) by dilution in nuclease-free water, 4% *w*/*v* trehalose and 28 mM histidine (pH 6.5). Before complexation, anti-ApoB (native and 2′Ome modified) and anti-GAPDH siRNA stock solutions were diluted to 0.2 mg/mL in the same buffer as chitosan and/or HA (0.83% trehalose and 5.83 mM histidine or 1% trehalose and 3.8 mM histidine).

### 2.4. Preparation, Lyophilization, and Reconstitution of Uncoated and HA-Coated Anti-ApoB Nanoparticles for the Assessment of In Vivo Toxicity

Uncoated and HA-coated anti-ApoB nanoparticles were prepared at a final N:P:C ratio of 5:1:0 and 2:1:1.5, respectively, using the advanced Automated In-line Mixing System (AIMS) as described before [7]. Chitosan at a specific N:P ratio (5:1 or 2:1) was mixed using a closed and sterile system comprising an LS14 Pharmapure tubing (1/16”) and two Masterflex L/S digital peristaltic pumps (Cole-Parmer, Montreal, QC, Canada), with siRNA (0.2 mg/mL) using a Y-connector and a mixing flow rate of 150 mL/min (Re = 4000). Anti-ApoB nanoparticles prepared at N:P = 2 were HA coated to a final N:P:C ratio of 2:1:1.5. Chitosan–siRNA nanoparticles (N:P:C ratio of 2:1:0) were mixed with HA at a 1:2 vol:vol ratio and a mixing flow rate of 150 mL/min (nanoparticles) and 75 mL/min for HA. Nanoparticles were incubated for 30 min at room temperature (RT) before analyses or freeze-drying. To inactivate possible nucleases, the whole closed system was treated with diethylpyrocarbonate (DEPC), autoclaved, and flushed with nuclease-free water.

Anti-ApoB nanoparticles were lyophilized under sterile conditions, using a 3-day cycle. Nanoparticle volumes of 2 and 5 mL were freeze-dried (FD) using a Laboratory Series Freeze-Dryer PC/PLC (Millrock Technology, Kingston, NY, USA). Samples were backfilled with Argon, stoppered, crimped, and stored at 4 °C until reconstitution. All freeze-dried samples were reconstituted to 12× initial concentration (208/417 μL to 5/10 mL serum vials respectively) and then incubated at RT for 5–10 min, and the concentration was adjusted by a nearly isotonic aqueous solution of 10% *w*/*v* trehalose and 70 mM histidine (pH 6.5) so that the desired dosage (mg siRNA/kg animal body weight) would be reached upon the injection of 10 μL of nanoparticle suspension per gram of body weight (BW).

### 2.5. Preparation of Uncoated and HA-Coated Anti-GAPDH Nanoparticles for Assessment of In Vivo Target Knockdown

Anti-GAPDH siRNA (0.2 mg/mL), low Mn chitosan (10 kDa), high Mn chitosan (120 kDa) and HA working solutions were prepared in the same way as described in the previous section. Uncoated chitosan–siGAPDH NPs were prepared at an N:P ratio of 5 by electrostatic mixing at a 1:1 vol:vol. HA-coated NPs were prepared at an N:P ratio of 2.5:1 by manual mixing (1:1 vol:vol), incubated at RT for 15 min, and coated with HA by mixing 1 part of HA working solution (0.4 mg/mL) to 2 parts of chitosan–siGAPDH NPs for a final N:P:C ratio of 2.5:1:2. The final volume never exceeded 1 mL, and chitosan was pipetted into siRNA. NPs were kept at RT for 20–30 min before administration to animals.

### 2.6. Preparation of Invivofectamine^®^-siRNA LNPs

Invivofectamine^®^ 2.0 and 3.0 were prepared as per the manufacturer’s recommendation. First, 250 μL of anti-ApoB siRNA (3 mg/mL) was diluted 1:2 in complexation buffer, mixed with 500 μL of Invivofectamine^®^2.0, vortexed for 30 s, incubated at 50 °C for 30 min, diluted with 14 mL of phosphate-buffered saline and concentrated at 4000 g using an Amicon Ultra-15 centrifugal filter unit (EDM Millipore Ltd., Etobicoke, ON, Canada) to a final volume of 872 μL (0.8 mg/mL siRNA).

For Invivofectamine^®^3.0, an anti-GAPDH siRNA (2.4 mg/mL) was mixed with a complexation buffer at 1:1 ratio and immediately added to Invivofectamine^®^3.0 at a 1:1 vol:vol ratio, vortexed for 30 s, incubated at 50 °C for 30 min and diluted to 0.25 mg/mL siRNA. All LNPs were subjected to QC (Dynamic Light Scattering (DLS), Doppler velocimetry, UV measurements and sterility assessment) and stored at 4 °C for 10–16 h before administration into mice. Invivofectamine^®^3.0 was used as replacement for Invivofectamine^®^2.0, which was discontinued at the time of the efficacy study.

### 2.7. Determination of Size and Surface Charge

The size and surface charge (ζ-potential) of NPs were determined by DLS and Laser Doppler velocimetry using a ZetaSizer Nano ZS device (Malvern Instruments Ltd., Malvern, UK). Measurements (N = 2–3, n = 6–9) were performed at a detector’s scattering angle of 173 at 25 °C using the viscosity of water as the sample diluent. NPs were diluted to 1× their initial concentration using NFW, which was followed by a dilution 1:4 and 1:8 using sterile 1% trehalose solution before determination of size and ζ-potential, respectively. A Smoluchowski equation was used to calculate the ζ-potential from the measured electrophoretic mobility.

### 2.8. Hemocompatibility

The hemolytic and hemagglutination properties of uncoated and HA-coated NPs were tested according to ASTM E2524-08 [32] and Evani et al. [31], respectively. The influence of dose, Mn, N:P ratio and HA coating on erythrocyte aggregation (hemagglutination) was investigated to better understand chitosan–blood interaction and limit potential in vivo toxicity. Blood was collected from healthy human donors following protocol approval by the Polytechnique Montreal Ethics Committee. Anti-ApoB NPs were prepared as described above, FD in the presence of 0.83% *w*/*v* trehalose, and 5.8 mM histidine (pH 6.5), and rehydrated to 12× the pre-FD concentration for the highest tested concentration (or dose) at iso-osmolality and then serially diluted to final siRNA concentrations of 0.1, 0.25, 0.5, and 0.8 mg/mL. Plasma-free hemoglobin (PFH) in the blood was measured at 0.49 mg/mL prior to assay. Total blood hemoglobin (TBH) was adjusted to a concentration of 10 ± 1 mg/mL (dTBH). NPs were diluted in dTBH at a 1:7:1 volumetric ratio with 100 μL of NPs at the target concentration pipetted into 700 μL PBS and 100 μL of blood (dTBH 10 ± 1 mg/mL). For colorimetric determination of hemolysis, 700 μL of samples was incubated at 37 °C for 3 h and visually inspected every 30 min for nanoparticle flocculation, dispersion, sinking or floating. Supernatant was collected following centrifugation at 800× *g* for 15 min, and absorbance was measured at 540 nm (Tecan Systems, Mannedorf, Switzerland). A four-parameter regression algorithm was used to obtain the calibration curve to calculate the hemoglobin concentration in the supernatant of each PFH sample. The percentage of hemolysis was computed as: *Hemolysis* (%) = 100 × (*PFHsample*/*dTBH*). For hemagglutination, the remaining 200 μL of each sample prepared above was pipetted in 96-well assay plates, incubated for 3 h, and visualized using an Axiovert light microscope, and the area covered by red blood cells was estimated and scored.

### 2.9. In Vivo Studies

In vivo experiments were randomized double blinded and approved by the University of Montreal Ethics Committee (CDEA) and the Montreal Heart Institute Research Center Ethics Committee. Mice (Charles River, Quebec, QC, Canada) were acclimatized in a pathogen-free facility with unrestricted access to water and food. Mice had body condition scores (BCSs) of 3 [31] with BW in the 20–25 g range at the time of injection. Injection volumes were calculated as 10 μL/g of BW and injections were performed within 10–15 s. Mice were euthanized by cardiac puncture followed by cervical dislocation.

### 2.10. Determination of Chitosan–siRNA Biodistribution Using Ex-Vivo Organ Imaging

Balb/c nude female (♀) mice aged 6 weeks weighing 20–22 g were used for biodistribution experiments. Test articles (naked siRNA, Invivofectamine^®^2.0 and chitosan-based NPs) formulated at an N:P:C ratio of 5:1:0 or 2:1:1.5 (Mn 10 and 120 kDa) were injected at a dose of 0.25 mg/kg DY647-labeled siRNA, except for the HA-coated NPs, which were administered at 0.165 mg/kg. DY647 fluorophore was administered at a dose of 0.5 mg/kg. Mice were euthanized 4 h post-administration and immediately perfused using PBS (1 × 20 mL) and 10% Neutral Buffer Formalin (NBF, 1 × 40 mL). Ex vivo imaging on collected organs was performed using a whole animal imaging system mounted with an EMCCD EM N2 camera (NUVU Cameras, Montreal, QC, Canada). Controls included PBS, naked DY647-labeled siRNA, DY647 alone, and commercially available lipid control Invivofectamine^®^2.0.

### 2.11. Determination of Chitosan–siRNA Nanoparticle In Vivo Toxicity

Unlike LNPs or liposomes, information on liver (or systemic) toxicity following the administration of uncoated (positively charged) and HA-coated (negatively charged) chitosan NPs is lacking. CD-1^®^ (ICR) female (♀) and male (♂) mice aged 4–5 weeks and weighing 22–24 g were administered test and control articles for toxicity study (7/group; 4 ♀ and 3 ♂). Mandibular blood was collected prior to and 4 h post-administration to prepare serum. Two out of seven mice from each group were euthanized at 4 h (1 ♀ and 1 ♂), and the remaining five (4 ♀ and 1 ♂) were euthanized 24 h post-administration. At each time point (4 versus 24 h), total circulating blood volume (tCBV) was collected by intra-cardiac puncture, and organs were harvested and washed in PBS. One half was immediately stored in liquid nitrogen (LiqN), and the second half was fixed in 10% NBF.

### 2.12. Hematological and Serological Parameters

The total circulating blood volume was split into lithium heparin and serum separation tubes, serum separated, for the comprehensive complete blood count and the “CC4” clinical chemistry panels using a Sysmex XTV 2000 (Sysmex, Mississauga, ON, Canada) and Beckman AU680 analyzers (Beckman Coulter Ltd., Mississauga, ON, Canada).

### 2.13. Determination of Cytokine Levels

Serum samples collected at 0 (baseline) and 4 h post-administration of test articles were assayed for pro-inflammatory cytokines (TNF-α, IL-1β, IL-6, KC and IFN-γ) using the Luminex^®^ technology. Plates were designed using the Bio-Plex^®^ assay builder (Bio-Rad Laboratories, Mississauga, ON, Canada), which was followed by the manufacturer’s QC. For each plate, a standard curve was prepared by diluting the Bio-Plex^®^ Pro Mouse Cytokine Standard 23-Plex in the Bio-Plex^®^ in standard diluent followed by 4-fold serial dilutions from 1:4 to 1:65536 in the same diluent. Samples were thawed on ice, cleared by centrifugation (10,000× *g*, 10 min, 4 °C), and diluted 1:4 using the Bio-Plex^®^ Sample diluent (Bio-Rad Laboratories, Mississauga, ON, Canada), and a volume of 20 μL was transferred to assay plates prefilled with pooled capture antibodies. The plates were incubated for 30 min under orbital shaking (800 rpm, RT), washed as per the manufacturer’s recommendation using a Bio-Plex^®^ Pro II Wash Station (Bio-Rad Laboratories, Mississauga, ON, Canada), incubated with biotinylated detection antibodies (30 min, 800 rpm, RT), washed and revealed post-incubation for 10 min with streptavidin–phycoerythrin (800 rpm, RT). Data were acquired on a Bio-Plex^®^ 200 system using the RP1 PMT setting (Bio-Rad Laboratories, Mississauga, ON, Canada) with a minimum of 50 beads per region analyzed. A standard curve was prepared using serial dilutions, and a 5-parameter regression algorithm was used to fit the data and interpolate each cytokine value in serum samples. To account for inter-plate variability, two samples (e.g., one LPS and one Invivofectamine^®^2.0 (8 mg/kg) sample) were used as inter-plate calibrators.

### 2.14. Determination of Chitosan–siRNA Nanoparticle In Vivo Efficacy

Balb/c male (♂) mice aged 6–7 weeks and weighing 22–25 g were used for an efficacy study. Uncoated anti-GAPDH NPs (92-10-5 and 92-120-5) and HA-coated NPs (HA92-10) were administered at 1 mg/kg (uncoated) and 8 mg/kg siRNA (HA-coated) every other day for a total of three injections. Naked anti-GAPDH siRNA (siGAPDH) and Altogen lipid NPs (Altogen LNP) were I.V. administered at 2.5 mg/kg every other day for a total of three injections. The liver-targeting Invivofectamine^®^3.0 lipid NPs (InvLNP) were I.V. injected at 2.5 mg/kg as a single injection. All mice were euthanized 72 h following the last administration to collect tCBV and organs. tCBV was serum separated, and organs were split into halves and stored in LiqN and fixed in 10% NBF before protein extraction, determination of GAPDH enzymatic activity, Western blotting, histology and immunohistochemistry.

### 2.15. Assessment of GAPDH Enzymatic Activity Using the KDalert^®^ Assay

Frozen tissues were cut on dry ice, weighed (~20 mg), and then disrupted using the 5 mm steel beads and TissueLyzer^®^ II system (Qiagen Inc, Toronto, ON, Canada) at 2 × 30 Hz, 20 s/cycle. Homogenized tissues were re-suspended in 750 μL of KDalert™ lysis buffer (Life Technologies, Burlington, ON, Canada) and incubated on ice for 30 min with inversions every 10 min. Lysates were clarified by centrifugation (2270× *g*, 30 min, 4 °C), transferred to new tubes, and diluted (1:20) in KDalert™ lysis buffer. A standard curve was prepared by diluting GAPDH stock solution (26 U/mL) with lysis buffer at a 1:100 ratio (GAPDH:Lysis), which was followed by 2-fold serial dilutions from 1:5 to 1:320. Twenty microliters of diluted samples, and standards, were transferred into 96-well plates and 180 μL of the KDalert™ Master Mix (Life technologies, Burlington, ON, Canada) was pipetted into each well. Plates were incubated for 15 min at RT, and absorbance was measured at 610 ± 10 nm using a TECAN Infinite^®^ F-500 microplate system (Tecan Systems, Mannedorf, Switzerland). The GAPDH activity was computed from the standard curve and normalized to the total protein content of the lysate sample as determined using the BioRad DC Protein assay kit (Bio-Rad Laboratories, Mississauga, ON, Canada).

### 2.16. Western Blotting

The affinity-purified monoclonal antibodies used were against GAPDH and vinculin. Kidney cortices were excised, homogenized using the TissueLyzer^®^ II system (Qiagen Inc., Toronto, ON, Canada) as described above, suspended in KD Alert lysis solution (Life technologies, Mississauga, ON, Canada), and centrifuged at 2270 g for 30 min at 4 °C. The supernatant was quantified using a BioRad DC Protein assay kit (Bio-Rad Laboratories, Mississauga, ON, Canada) and diluted in SDS buffer containing a final concentration of 62 mM Tris (hydroxymethyl)-aminomethane, 0.1 M SDS, 8.7% glycerol, 0.09 mM bromophenol blue, and 0.04 M dithiothreitol (DTT). The samples were heated for 5 min at 90 °C, loaded into Protean mini TGX SDS-PAGE (4–12%) gradient polyacrylamide gels (Bio-Rad Laboratories, Mississauga, ON, Canada), and overnight wet transferred to Amersham™ HyBond^®^ P PVDF membranes (GE Lifesciences, Mississauga, ON, Canada). Membranes were dried and blocked for 1 h at room temperature in 5% non-fat milk, probed overnight at 4 °C with anti-GAPDH primary antibody (1:1000), washed (3X, 15 min, 1% Triton in the presence of blocking buffer), and incubated with HRP-conjugated anti-rabbit IgG1 secondary antibody (1:500) for 1 h, washed, revealed using the Clarity Max™ ECL substrate (Bio-Rad Laboratories, Mississauga, ON, Canada) and visualized using the ChemiDoc MP™ system (Bio-Rad Laboratories, Mississauga, ON, Canada). Protein band quantification was performed using ChemiDoc MP software.

### 2.17. Clinical Signs and Body Weight

Mice clinical signs were determined for 4 h post-administration of test articles and at euthanasia. Scores for clinical signs—body condition, general aspect, natural behavior, and provoked behavior—were recorded by trained personnel and qualified animal care technicians. The Mouse Grimace Scale (MGS) was also used for the scoring of clinical signs in case of distress. Bodyweight was recorded prior to each injection and at euthanasia and expressed as a percent change relative to the previous injection.

### 2.18. Histology and Immunohistochemistry

Samples were fixed in 10% NBF, embedded in paraffin to collect 5 μm sections followed by hematoxylin and eosin staining and immunohistochemical analysis of GAPDH (Ab181602, 1:250 dilution). Prior to immunohistochemistry, antigen retrieval was performed with 10 mM Tris/1 mM EDTA pH 9 at 60 °C. Sections were blocked with 20% (*v*/*v*) goat serum/0.1% (*v*/*v*) Triton X-100/PBS for 1 h at room temperature and then incubated for 16 h at 4 °C with Rabbit monoclonal anti-GAPDH diluted 1:250 in 10% (*v*/*v*) goat serum/0.1% (*v*/*v*) Triton X-100/PBS. Sections were then incubated for 1 h at room temperature with biotinylated goat anti-Rabbit IgG (Ab97049) diluted 1:500 in 10% (*v*/*v*) goat serum/0.1% (*v*/*v*) Triton X-100/PBS. Revelation was performed with the Vectastain Avidin Biotin Complex (ABC)–alkaline phosphatase (ALP) and AP Red substrate kits (Vector Laboratories, Burlingame, CA, USA). Sections were counterstained with a Weigert Iron Hematoxylin prior to dehydration, clearing and mounting. Slides were scanned using a NanoZoomer digital slide scanner (Hamamatsu, Boston, MA, USA) and visualized using the NDP^®^ view 2.0 software (Hamamatsu, Boston, MA, USA).

### 2.19. Confocal Laser Scanning Microscopy

For the in vivo biodistribution and subcellular localization of DY647-labeled siRNA, organs were cryosectioned (5 μm), actin stained using AlexaFluor 546 phalloidin and mounted with ProLong^®^ Diamond antifade containing DAPI. Sections were imaged in multitrack mode using a Zeiss LSM 510 META confocal Axioplan 200 microscope (Carl Zeiss AG, Feldbach, Switzerland).

### 2.20. Statistical Analysis

Data were collected and expressed as average ± standard deviation. Statistical analysis was conducted using a GraphPad Prism^®^ 7.0 (GraphPad Software Inc., La Jolla, CA, USA) software package. Unless otherwise stated, one-factor ANOVA followed by Dunnet’s test for multiple comparisons was performed on collected data.

## 3. Results

### 3.1. Characterization of Injected NPs

**Figure 1:**
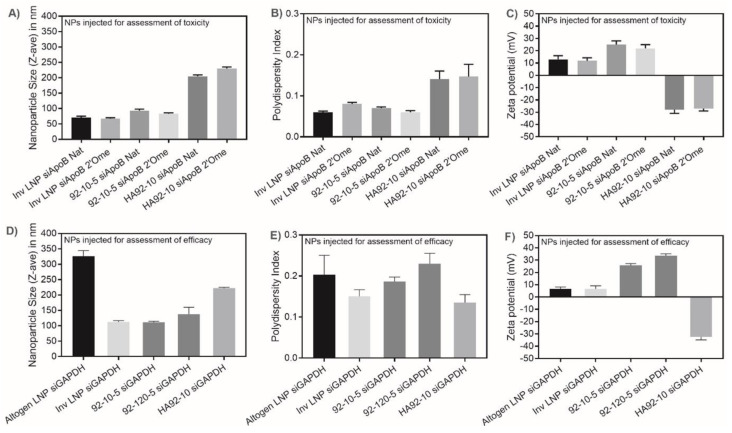
(**A**) Size (Z-average diameter in nm), (**B**) polydispersity index (PdI), and (**C**) surface charge (ζ-potential) of NPs injected for the assessment of toxicity. (**D**) Size (Z-average diameter in nm), (**E**) polydispersity index (PdI), and (**F**) surface charge (ζ-potential) of NPs injected for the assessment of in vivo knockdown efficacy. Size, polydispersity index and surface charge (ζ-potential) of chitosan-based siRNA nanoparticles and LNPs. InvLNP: Invivofectamine^®^ 2.0 were formulated using the AIMS with unmodified (siApoB Nat) or 2′O-methyl modified anti-ApoB siRNA (2′Ome siApoB) sequences (panels (**A**–**C**)). Altogen LNPs and InvLNP: Altogen and Invivofectamine^®^ 3.0 were manually formulated with LNA-modified anti-GAPDH siRNA (panels (**D**–**F**)). 92-10-5: Low molecular weight chitosan, with a degree of deacetylation of 92% and molecular weight (Mn) of 10 kDa (92-10), was formulated with siApoB Nat or 2′Ome siApoB at an amine-to-phosphate ratio (N:P ratio) of 5 (panels (**A**–**C**)). 92-10-5 and 92-10-120: Low Mn (10 kDa) and high Mn (120 kDa) chitosans were formulated with LNA modified anti-GAPDH siRNA at an N:P ratio of 5 (panels (**D**–**F**)). HA (866 kDa)-coated chitosan NPs (HA92-10) were prepared at an N:P ratio of 2 and coated with HA at a phosphate-to-carboxyl ratio (P:C) of 1.5 (panels (**A**–**F**)). The size, PdI and ζ-potential of LNPs were measured in phosphate-buffered saline (PBS, pH 7.4). The size, PdI and ζ-potential of uncoated and HA-coated chitosan–siRNA NPs were measured in excipients (1% trehalose (*w*/*w*), 5.8 or 3.5 mM histidine, pH 6.5). Data represent the average ± standard deviation of 3 independent experiments with 2 technical replicates per experiment (N = 3, n = 6).

Lipid and chitosan-based NPs were in the range of 60–100 nm with HA coating increasing chitosan NP size by two-fold (Figure 1A). The polydispersity index (PdI) was below 0.2, indicating homogenous particles. Chitosan-based NPs were positively charged with a ζ-potential between 25 and 30 mV. The HA coating at an N:P:C ratio of 2:1:1.5 inversed the surface charge to around −30 mV. InvLNPs were quasi-neutral (~8–10 mV). siRNA composition and chemical modification had no impact on the NP physicochemical characteristics (Figure 1). Polymer length (Mn) and mixing regimen influenced surface charge and PdI, respectively (Figure 1A,B,C vs. Figure 1D,E,F).

### 3.2. Uncoated Chitosan NPs Induced Hemolysis and Hemagglutination at High Doses Which Were Abrogated by HA Coating

**Figure 2:**
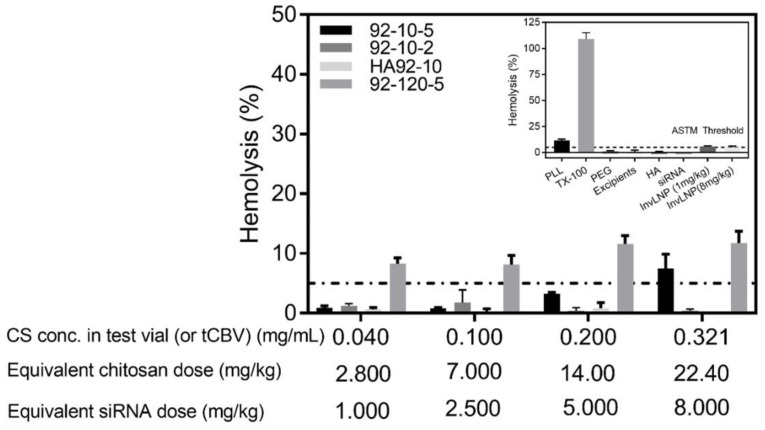
Hemocompatibility profiling of uncoated and HA-coated chitosan–siRNA NPs via red blood cell (RBC) lysis. Low (10 kDa) versus high (120 kDa) molecular weight chitosans were formulated with HPLC-grade siRNA. HA-coated formulations were formulated at an N:P:C ratio of 2:1:1.5. Increasing doses of siRNA were mixed with human pooled blood and % hemolysis determined as per ASTM-E2524-08 [32]. The concentration of chitosan (mg/mL) in the test vial (equivalent to the concentration in total circulating blood volume or tCBV), the equivalent chitosan dose in mg/kg of body weight and the corresponding siRNA dose in mg/kg for N:P of 5 are shown. Inset shows data from positive and negative controls. Poly-L-Lysine (PLL), Triton-X-100 (TX-100), polyethylene glycol (PEG), excipients (1% trehalose, 5.8 mM histidine, pH 6.5), HA 866 kDa, siRNA (8 mg/kg) and Invivofectamine^®^ 2.0 (1 versus 8 mg/kg of siRNA). Data represent the average ± standard deviation of 2 independent experiments with 3–6 technical replicates per experiment (N = 2, n = 6–12). In the figure legend, 92 refers to chitosan DDA, 10 or 120 refer to chitosan target Mn and 2 or 5 refer to the N:P ratio.

A dose-dependent increase in hemolysis was observed for both low (10 kDa) and high (120 kDa) Mn chitosan (Figure 2). Erythrocyte lysis was abolished with a reduction in free chitosan by reducing N:P 5 to 2 and by HA coating. A two-fold increase in hemolysis was observed with an increase in siRNA dose or chitosan concentration in blood from 0.040 to 0.321 mg/mL indicating a non-linear relationship for high Mn chitosan. Negative controls (PEG and HA) were within the ASTM standard [32] (Figure 2, Inset), whereas excipients (buffer) and siRNA were found to be non-hemolytic.

InvLNP assayed at 1 and 8 mg/kg siRNA showed around 5% hemolysis with no dose effect (Figure 2, Inset) indicating minimal interaction with blood erythrocytes at pH 7.4. Supplemental Figure S1 shows both low and high Mn chitosan induced dose-dependent hemagglutination above a threshold of 1 mg/kg siRNA.

### 3.3. Uncoated and HA.-Coated Chitosan NPs Promoted Extrahepatic Delivery of siRNA to Kidney Proximal Tubular Epithelial Cells (PTEC)

**Figure 3:**
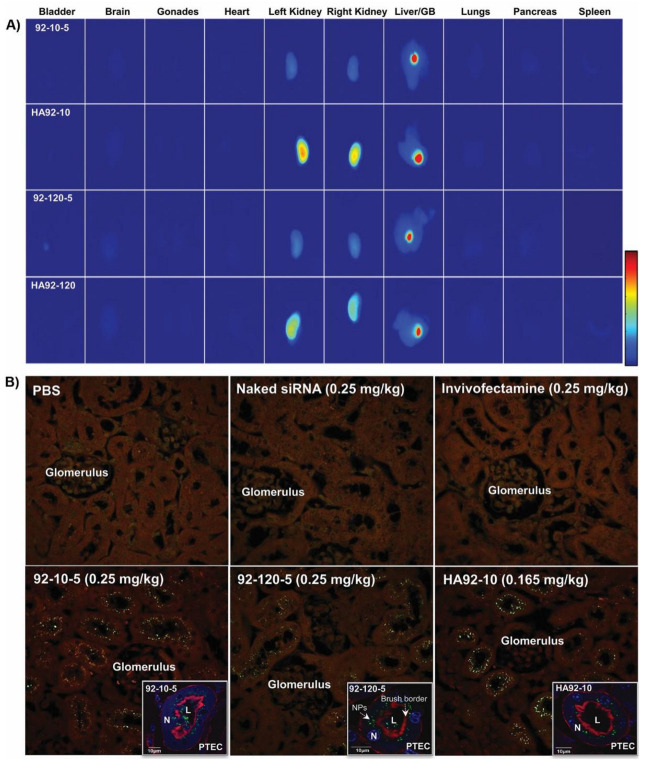
In vivo biodistribution of uncoated and HA-coated chitosan–siRNA NPs. (**A**) Effect of Mn and HA coating on the biodistribution of chitosan–siRNA NPs. Uncoated NPs were injected in Balb/c nude mice at a dose of 0.25 mg/kg of DY647-labeled siRNA (equivalent dose of 0.7 mg/kg of chitosan), HA-coated NPs were injected at a dose of 0.165 mg/kg of DY647-labeled siRNA (equivalent dose of 0.2 mg/kg of chitosan) and organs were imaged ex vivo 4 h post-administration. (**B**) Histological and CLSM images of NPs accumulated in PTEC. NPs were injected as described above, organs perfused and collected 4 h post-administration, fixed and cryo-sectioned (5 µm). For CLSM insets, sections were stained with phalloidin red and DAPI. (PBS) phosphate-buffered saline, (siNaked) naked DY647-labeled siRNA, (Invivofectamine) LNPs, (PTEC) proximal epithelial tubular cells, (NPs) nanoparticles, (L) lumen. DY647 siRNA = green, nucleus (N) = blue, and brush borders = red (actin staining).

Chitosan-based NPs accumulated in the kidney and gallbladder (Figure 3A), and the HA coating of the NPs increased siRNA accumulation in the kidney and gallbladder without altering the bio-distribution profile observed with uncoated NPs. Controls, InvLNP, naked siRNA, and DY647 alone, accumulated in the liver and spleen (InvLNP), kidney (naked siRNA), and bladder (DY647), respectively (Supplemental Figure S2). The fluorescent signal intensity of naked siRNA in the kidney was several folds lower compared to chitosan and HA-coated chitosan NPs (Figure 3A vs. Supplemental Figure S2).

Histological sections were examined under CLSM to examine the cellular and subcellular localization of the delivered siRNA. siRNA formulated in NPs accumulated predominantly in the proximal tubule epithelial cells (PTECs) independently of chitosan Mn and HA coating (Figure 3B). The siRNA accumulation in PTECs was greatly enhanced with the HA coating, which was indicated by an increase in fluorescence at a lower dose of 0.165 vs. 0.25 mg/kg for uncoated NPs. Actin staining using phalloidin red revealed a typical punctuate siRNA pattern across the brush border membrane lining the PTEC, indicating intracellular localization (Figure 3, Insets). In contrast to LNPs, chitosan-based NP accumulation in the kidney represents a new approach to treat PTEC-dependent pathologies.

### 3.4. Unlike Cationic LNPs, Uncoated and HA-Coated Chitosan NPs Did Not Induce Immune Stimulation and Hematologic Toxicity upon Intravenous Administration

**Figure 4:**
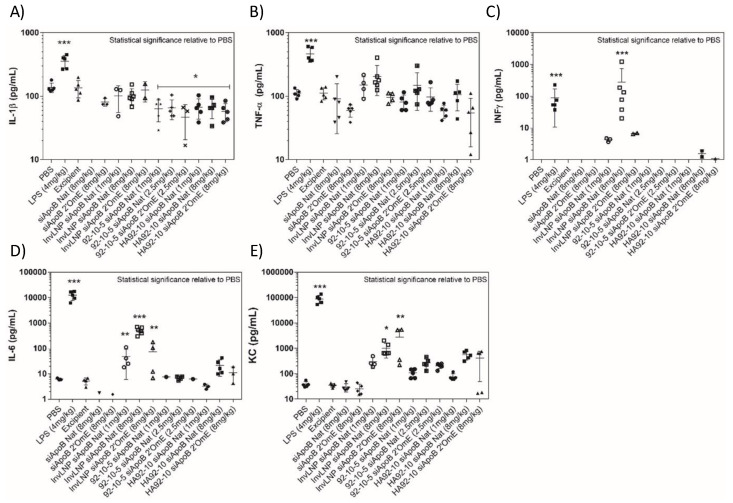
Cytokine induction 4 h post-injection of a single ascending dose of Invivofectamine^®^ 2.0, uncoated and HA-coated chitosan siRNA NPs in CD-1^®^ (ICR) mice. In the figure, PBS = phosphate-buffered saline, LPS = lipopolysaccharide, InvLNP = Invivofectamine^®^ 2.0-siRNA LNPs, siApoB Nat = unmodified anti-ApoB siRNA sequence, siApoB 2′Ome = 2′O methyl modified anti-ApoB siRNA sequence, 92-10-5 = Chitosan 92% DDA target Mn 10 kDa N:P ratio 5, and HA = hyaluronic acid 866 kDa. Mice were I.V. injected with test articles, serum was collected and analyzed 4 h post-injection using the BioPlex™ 200 system. Each symbol represents an animal and data represent average values ± standard deviation of 5–7 animals. Statistical significance versus PBS-treated animals was computed with one-way ANOVA followed by Dunnett’s test for multiple comparisons: * *p* < 0.01, ** *p* < 0.001, *** *p* < 0.00001. Note: In order to not bias the average, cytokine levels (animals) below the range of detection (< OOR) were excluded and not considered as 0 or lower limit of quantification (LLOQ) (pg/mL).

Pro-inflammatory type-I cytokines (IL-1β, TNF-α, INFγ, IL-6 and KC) measured in serum 4 h post-injection were markedly increased by bacterial LPS and InvLNPs (Figure 4). No significant induction was observed with uncoated and HA-coated NPs. InvLNPs showed a dose-dependent significant induction of INFγ, IL-6 and KC and a minor TNF-α increase in serum. Chemical modification (2′Ome) of the uridine (U) and guanine (G) nucleotides of the anti-ApoB siRNA (siApoB 2′Ome) abolished cytokine induction except KC (CXCL1). In contrast, uncoated and HA-coated chitosan did not significantly induce any of the assayed pro-inflammatory cytokines, demonstrating low in vivo immune stimulating potential following I.V. injection of chitosan-based systems. A small but significant reduction in IL-1β was observed with all chitosan formulations (Figure 4).

Although the decrease in IL-1β was only observed with chitosan, pre- vs. post-injection levels of IL-1β and TNF-α showed no significant changes and were generally lower than the PBS and excipient groups (Figure 4 and Supplemental Figure S3). Pre- vs. post-CS injection (4 h) showed a two-fold but statistically insignificant increase in KC.

**Figure 5:**
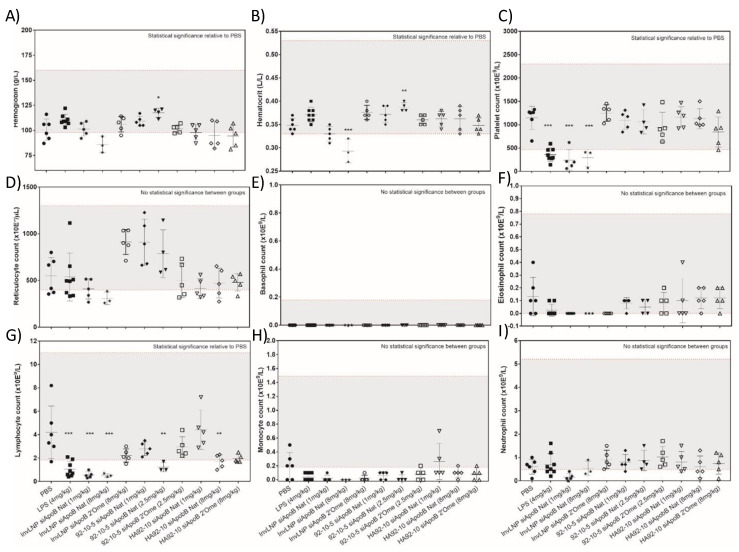
Hematological profiling of Invivofectamine^®^ 2.0, uncoated and HA-coated chitosan siRNA NPs following single ascending dose administration in CD-1^®^ (ICR) mice. In the figure, PBS = phosphate-buffered saline, LPS = lipopolysaccharide, InvLNP = Invivofectamine^®^ 2.0-siRNA LNPs, siApoB Nat = unmodified anti-ApoB siRNA sequence, siApoB 2′Ome = 2′O methyl modified anti-ApoB siRNA sequence, 92-10-5 = Chitosan 92% DDA target Mn 10 kDa N:P ratio 5, and HA = hyaluronic acid 866 kDa. Mice were intravenously injected with test articles, blood collected and analyzed 24 h post-injection at IDEXX laboratories. Each symbol represents an animal and lines represent average values ± standard deviation of 5–7 animals except for InvLNP siApoB Nat (8 mg/kg) where 3 animals were assayed for hematology. The gray shaded area represents the normal values (95% confidence interval, N = 266 divided as 133 ♀ and 133 ♂) of 8–12 week old CD-1^®^ (ICR) mice from Charles River Laboratories (North American colonies). Statistical significance versus PBS-treated animals was computed with one-way ANOVA followed by Dunnett’s test for multiple comparisons: * *p* < 0.01, ** *p* < 0.001, *** *p* < 0.00001. Note: Normal range limits in this figure are not firm boundaries and should be used as guidelines, since a large range of values was reported in the literature and could be accounted for by variation in age, sex, sampling technique and testing methodology (i.e., instrument, technique etc.).

Hemoglobin and hematocrit levels decreased with high doses of LNPs (8 mg/kg) and increased with uncoated chitosan NPs at high dose (2.5 mg/kg) with no correlation to the absolute reticulocyte count (Figure 5). Platelet counts decreased significantly with both LPS and InvLNPs encapsulating the native ApoB sequence (siApoB Nat) indicating acute thrombocytopenia (decreased platelet counts). The use of chemically modified siRNA (siApoB 2′Ome) abrogated the sharp decline in platelets (Figure 5). Unlike LNPs, no sequence or vector-dependent thrombocytopenic effect was observed with uncoated and HA-coated chitosan NPs (Figure 5). LPS, InvLNPs, high doses of uncoated (2.5 mg/kg) and HA-coated (8 mg/kg) chitosan-based NPs decreased the circulating lymphocyte count (Figure 5). However, the effect of chitosan-based NPs in decreasing the lymphocyte count was weaker than lipid NPs with values at the lower limit of the CD-1^®^ (ICR) normal reference values (Figure 5).

### 3.5. Liver and Kidney Biomarkers Remain Unchanged with Uncoated and HA-Coated Chitosan NPs While High Doses of Lipid NPs Led to Increased Transaminase Levels

**Figure 6:**
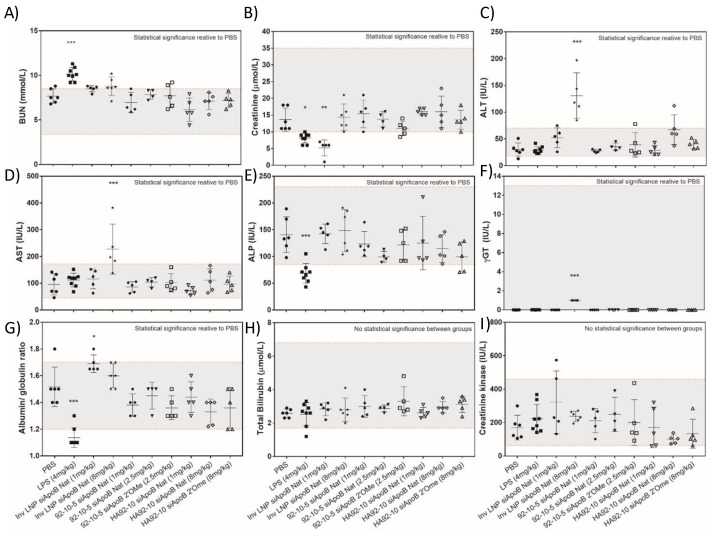
Serological profiling of Invivofectamine^®^ 2.0, uncoated and HA-coated chitosan siRNA NPs following single ascending dose administration in CD-1^®^ (ICR) mice. In the figure, PBS = phosphate-buffered saline, LPS = lipopolysaccharide, InvLNP = Invivofectamine^®^ 2.0-siRNA LNPs, siApoB Nat = unmodified anti-ApoB siRNA sequence, siApoB 2′Ome = 2′O methyl modified anti-ApoB siRNA sequence, 92-10-5 = Chitosan 92% DDA target Mn 10 kDa N:P ratio 5, and HA = hyaluronic acid 866 kDa, BUN (blood urea nitrogen), ALT (alanine transaminase), AST (aspartate transaminase), ALP (alkaline phosphatase), γGTT (gamma glutamyl transferase). Mice were intravenously injected with test articles, blood collected and analyzed 24 h post-injection at IDEXX laboratories. Each symbol represents an animal and data represent average values ± standard deviation of 5–7 animals except for InvLNP siApoB Nat (8 mg/kg) where 3 animals were assayed for hematology. The gray shaded area represents the normal values (95% confidence interval, N = 266 divided as 133 ♀ and 133 ♂) of 8–12-week-old CD-1^®^ (ICR) mice from Charles Rivers Laboratories (North American colonies). Statistical significance versus PBS-treated animals was computed with one-way ANOVA followed by Dunnett’s test for multiple comparisons: * *p* < 0.01, ** *p* < 0.001, *** *p* < 0.00001. Note: Normal range limits in this figure are not firm boundaries and should be used as guidelines, since a large range of values was reported in the literature and could be accounted for by variation in age, sex, sampling technique and testing methodology (i.e., instrument, technique etc.).

Blood urea nitrogen (BUN) and creatinine (Cr) were within the normal reference ranges and comparable to the PBS group following injection with uncoated and HA-coated chitosan-based NPs (Figure 6). Chitosan-based NPs targeting kidney PTEC (Figure 6) appeared to be well tolerated for at least 24 h post-injection with no changes in kidney biomarkers. An increase in BUN with a concomitant decrease in Cr was observed for the LPS-treated group, which was consistent with increased protein catabolism, reduced clearance, and the induction of cytokines (Figure 6) associated with fever-like symptoms or infections. Surprisingly reduced Cr with normal BUN was observed with Invivofectamine^®^ 2.0-siRNA LNP (InvLNP siApoB Nat) only at a low dose. ALT, AST and ALP levels were within the normal range and comparable to the PBS control 24 h post-administration of chitosan-based NPs (Figure 6). InvLNPs demonstrated a dose-dependent increase in liver biomarkers (Figure 6) with a 2 to 3-fold increase in the ALT/AST ratio. γ-glutamyl transferase (γGT), a relevant biomarker for liver and bile duct injury, total bilirubin (TBil) and creatine kinase (CK), a biomarker for muscle toxicity, were within the normal range for all formulations. LPS induced a decrease in ALP and albumin/globulin ratio with no effect on ALT, AST, γGT, TBil and CK.

### 3.6. Despite Normal Clinical Signs Post-Administration of NPs, a Decrease in Body Weight Was Observed with Cationic Lipid NPs, Specifically Following Multiple Injections

**Figure 7:**
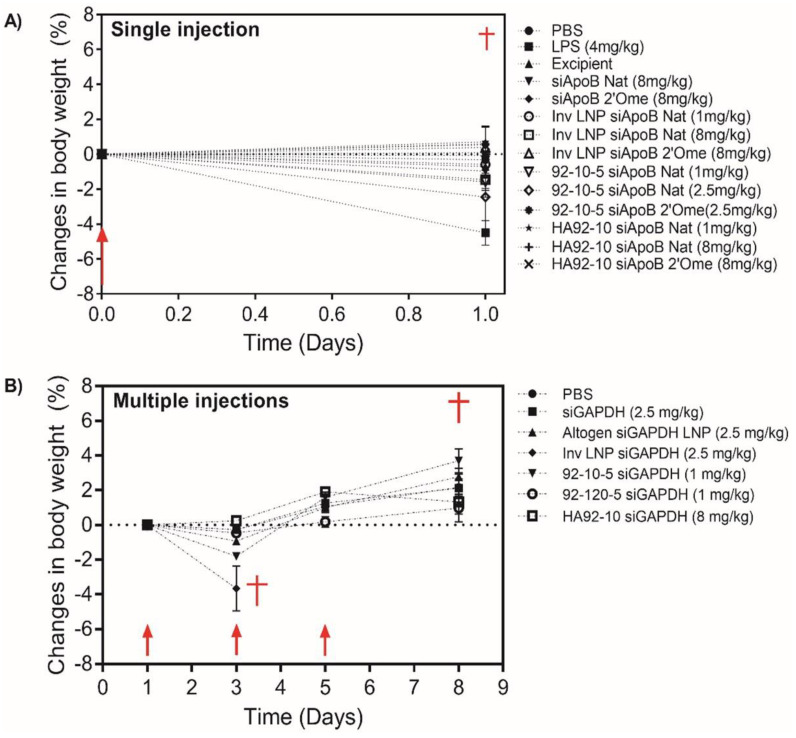
Changes in body weight following intravenous nanoparticle administration. (**A**) Percent change in body weight following a single intravenous injection in CD-1^®^ (ICR) mice. InvLNPs were formulated with unmodified (siApoB Nat) and 2′O-methyl modified ApoB siRNA (siApoB 2′Ome) and injected at 1 and 8 mg/kg. Uncoated chitosan was formulated with siApoB Nat and siApoB 2′Ome at an N:P ratio of 5 and injected at 1 and 2.5 mg/kg. HA (866 kDa)-coated NPs were prepared at an N:P:C ratio of 2:1:1.5 and injected at 1 and 8 mg/kg. The injected doses were chosen from the hemocompatibility data (Figure 2) where the maximum dose results in hemolysis below the ASTM threshold. (**B**) Percent change in body weight following three I.V. injections in Balb/c mice. InvLNP and Altogen (Altogen LNP) were formulated with LNA-modified GAPDH siRNA (siGAPDH) and injected at 2.5 mg/kg. Low Mn (10 kDa) and high Mn (120 kDa) chitosan NPs were formulated with siGAPDH at an N:P ratio of 5 and I.V. injected at 1 mg/kg. HA (866 kDa)-coated NPs were prepared at an N:P:C ratio of 2:1:1.5 and injected at 8 mg/kg. The injected doses were chosen from the hemocompatibility data (Figure 2) where the maximum dose results in hemolysis below the ASTM threshold and following personal communication with the manufacturers of Invivofectamine^®^ 3.0 and Altogen. For panels (**A**,**B**), body weight (g) was collected before each injection and at euthanasia. Red arrows and crosses illustrate injection and euthanasia, respectively. Data represent the average ± standard deviation of 5–7 mice/group. Phosphate-buffered saline (PBS) and lipopolysaccharide (LPS) were used as controls.

Next, we monitored clinical signs, body weights and gross organ pathology to assess toxicity following single and multiple injections (Figure 7 and Table S1) of uncoated, HA-coated and LNPs. A single I.V. injection led to a small but statistically insignificant decrease in BW (~0–2%) for quasi-neutral InvLNPs (ζ-potential ~ 11 ± 3 mV), HA-coated NPs (ζ-potential ~ 25 ± 5 mV) and low doses (1 mg/kg) of uncoated NPs (ζ-potential ~ 25 ± 5 mV) compared with PBS, excipient and naked siRNA groups that showed a steady, or slight (<1%) increase in BW (Figure 7). The LPS-treated group showed a sharp decline in BW (4 ± 0.5%) (Figure 7). The sharp decrease in BW correlated with clinical signs (Table S2) where LPS-injected mice showed signs of lethargy, delayed responsiveness to stimuli and changes in their general appearance around 4 h post-injection with decreased ALP levels (Figure 6).

Multiple dosing with uncoated and HA-coated chitosan NPs was well tolerated with no decrease in body weight observed following the injection of low (10 kDa) or high Mn (120 kDa) formulations. In contrast to CS-based NPs, Invivofectamine^®^ 3.0 induced a BW decrease of 4 ± 1%.

### 3.7. Uncoated Chitosan NPs Demonstrated Functional Gene-Specific Knockdown in Kidney Cortex Independent of Polymer Length (Mn)

**Figure 8:**
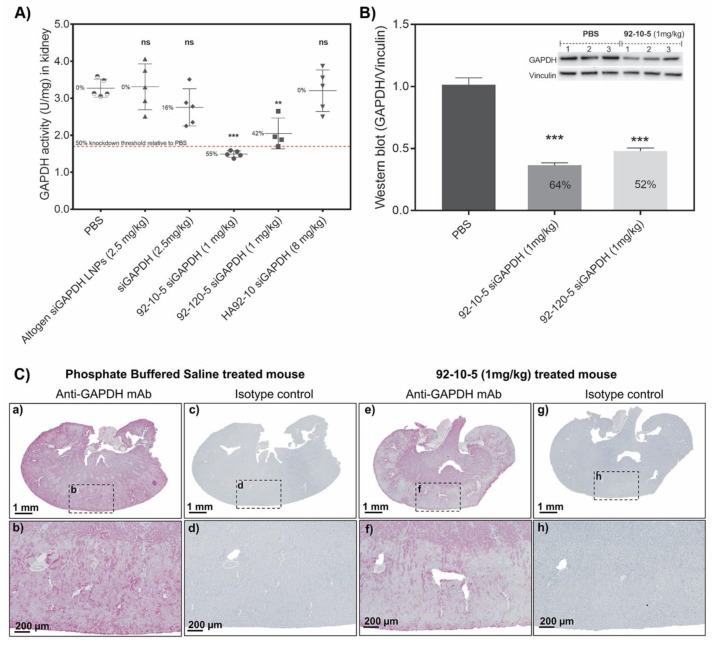
Efficacy of in vivo target knockdown. (**A**) GAPDH activity (U) normalized per tissue mass (mg). Kidneys were collected, snap frozen in liquid nitrogen, and cortex excised, homogenized, protein extracted and assayed using the GAPDH KDalert™ enzymatic kit. (ns) non-significant and numbers express % knockdown relative to PBS. (**B**) Western blot detection of GAPDH in kidney lysate. GAPDH signal was normalized to the vinculin loading control. Inset shows an actual example of a Western blot membrane used for quantification. The membrane shows three different animals injected with PBS (control) and with 92-10-5 (1 mg/kg siGAPDH). Numbers in the histogram columns represent % knockdown relative to PBS. (**C**) Qualitative assessment of GAPDH knockdown in kidney by immunohistochemistry. Panels (**a**–**d**) show a kidney section collected from a PBS-treated animal, stained with anti-GAPDH antibody (**a**,**b**) and isotype control (**c**,**d**). Panels (**e**–**h**) show a kidney section collected from a chitosan (92-10-5)-treated animal, stained with anti-GAPDH antibody (**e**,**f**) and isotype control (**g**,**h**). Data represent average values ± standard deviation of 5 animals except for 92-120-5 siGAPDH (1 mg/kg) where 4 animals were assayed. Statistical significance versus PBS-treated animals was computed with one-way ANOVA followed by Dunnett’s test for multiple comparisons: * *p* < 0.01, ** *p* < 0.001, *** *p* < 0.00001.

A significant functional knockdown of 55% assessed by GAPDH enzymatic activity (Figure 8A) was achieved in the kidney following the administration of 1 mg/kg of uncoated chitosan NPs. Low (10 kDa) and high (120 kDa) Mn chitosan achieved similar knockdown efficiency with slightly improved performance observed with low Mn chitosan (Figure 8A,B). Assessment of GAPDH knockdown by Western blot showed a similar knockdown trend between low versus high Mn chitosan with minor differences. HA-coated formulation, injected at the higher dose of 8 mg/kg, produced no knockdown of the target gene. In our study, chemically modified siRNA, containing a locked nucleic acid combined with other AMBION Silencer^®^ Select modifications (undisclosed), resulted in only 16% target knockdown when injected at a dose of 2.5 mg/kg indicating an improved siRNA knockdown efficiency of nearly 4-fold to 55% by the chitosan delivery system. Surprisingly, the commercially available kidney targeted liposome, Altogen LNP, did not achieve knockdown. The qualitative confirmation of target knockdown using immunohistochemistry showed target-specific knockdown in the cortex of chitosan NP-treated kidneys (Figure 8C).

## 4. Discussion

Here, we investigated the effect of chitosan polymer length, dose, and surface modification with HA on the hemolytic potential, acute and organ toxicity, cytokine induction, in vivo biodistribution and target knockdown efficacy in addition to comparing chitosan NPs with commercially available cationic LNPs (Invivofectamine^®^). Taken together, our data showed that uncoated chitosans (high and low Mn, 92% DDA) are safe, well tolerated, non-immune stimulating delivery systems that target kidney PTECs to achieve significant functional knockdown in kidney cortices.

Hemolytic and hemagglutination properties have been well characterized for cationic polymers such as PEI [33,34] and chitosan [10] with chitooligosaccharide (Mn < 5 kDa) found to be non-hemolytic but causing dose-dependent erythrocyte aggregation [35]. Additionally, our previous in vitro study demonstrated the non-genotoxic effect of HA-coated chitosan NPs [36]. Here, we show that uncoated chitosan NPs display dose- and molecular weight-dependent hemolytic and hemagglutination properties that could be abrogated with the use of NPs prepared at a low N:P ratio or HA coating (Figure 2), highlighting careful dosing to avoid hemotoxicity and/or embolism. The maximum siRNA dose that could potentially be intravenously administered with chitosan was found to depend on the Mn, N:P ratio and HA coating. According to PEGylated LNPs standard, a hemolytic index below 5% is regarded safe [34]. Consequently, doses of 5 and 1 mg/kg siRNA could be administered with low and high Mn chitosan, respectively, when formulated at N:P 5, while doses of at least 8 mg/kg siRNA could be used for N:P 2 and HA-coated NPs (Figure 2). The hemolytic/hemagglutination potential of chitosan could occur through the interaction with negatively charged erythrocyte (RBC) membranes via a pore-forming mechanism, followed by an osmotic shock, and/or through the regulation of the surface protein and increase in surface roughness, as demonstrated before [35]. Moreover, the interaction between chitosan amino and acidic groups on erythrocytes could promote polyelectrolyte complex formation causing RBC aggregation as seen for other biomaterials [37]. NP coating with HA, a biocompatible and negatively charged molecule, eliminated both hemolysis and RBC aggregation possibly due to limited interaction with erythrocyte membranes through electrostatic repulsion and reduced interaction with serum components. Unlike uncoated chitosan, LNPs did not show dose dependent hemolysis, which was probably due to surface PEGylation implied by the quasi-neutral ζ-potential ~ 8–10 mV. Shielding with PEG has been the method of choice to limit LNP hemolysis with high PEG density required for improved biocompatibility and reduced cytokine induction [38] and is incorporated in most LNPs available commercially or in clinical development. Although the exact composition of InvLNPs is not disclosed by the manufacturer, a quasi-neutral surface charge is probably associated with PEGylation or an increased molar ratio of neutral to cationic lipids in the formulation. Unlike PEGylation, electrostatic coating with HA demonstrated a similar protective effect, permitting a dose increase to at least 8 mg/kg.

The immune-stimulating properties of NPs, or their payloads, monitored through the expression of cytokines in plasma, serum or target tissues [39,40,41,42] represent a major hurdle for clinical translation. Our uncoated and HA-coated chitosan NPs did not induce type-I pro-inflammatory cytokines (IL-1β, TNF-α, INFγ and IL-6) except for a small, statistically insignificant increase in KC, which is a human IL-8 homologue indicating a non-immunogenic effect 4 h post-administration. Since KC has distinct target specificity for neutrophils [43,44], the absence of neutrophil invasion, 24 h post-administration, in organs where chitosan had accumulated suggests an epithelial cell-independent mechanism of KC expression.

The adjuvant and immune stimulating effect of CS involves the activation of DCs and the secretion of type-I cytokines through NLRP3 inflammasome activation and the recently discovered cGAS/STING pathway for lower DDA (80%) chitosans [11,12,45,46,47]. An apparent contradiction between the lack of cytokine activation here and the literature could be explained by differences in routes of administration, dose, DDAs and priming of immune cells. For instance, most studies demonstrating the anti-allergic properties of chitin and chitosan (Th2 inhibition) via the expression of type-I cytokines have been tested in vitro and/or using the intranasal, intraperitoneal, intraocular and intravaginal routes of administration [12,45]. However, in all these studies, priming strategies were used and could explain cytokine induction consistent with the finding that chitosan stimulated significant cytokine release only from primed BMMΦ [46]. Here, we did not measure cytokine levels at subsequent time points, which could also explain the absence of cytokine induction, which only appeared around 9 h and peaked 24 h post-stimulation [12]. Other considerations such as Mn, contaminants, particle size may also contribute to the observed difference.

LNPs and liposomes possess potent immune stimulation governed by the lipid and cationic head groups and/or the combination with the nucleic acid payload [38,39,40,41,48]. In this study, Invivofectamine^®^ LNPs demonstrated a dose-dependent induction of INFγ, IL-6 and KC and a minor TNF-α increase in serum. Immune stimulation was abrogated by 2′Ome-modified siRNA, confirming previous results with LNPs [39,40] highlighting major differences with our chitosan system where cytokine induction was not observed with any payload. Since TNF-α—a potent cytokine—is activated by the activation of Toll-Like receptors (TLRs) [49,50], the TNF-α stimulation observed with LNP used in this study, while not with chitosan, suggests a TLR-based mechanism of immune induction reminiscent of Chol:DSPC:DOTAP (3:1:1) cationic liposomes [41].

We then examined the acute toxic effects of chitosan, dose, siRNA sequence, and HA coating on hematological and serological parameters. Hematocrit (HCT) and total hemoglobin (Hb) levels were unchanged versus PBS and within the normal reference ranges of CD-1^®^ (ICR) mice, indicating a relatively safe and non-hemolytic profile for all formulations tested. Lower Hb but not HCT levels compared with the reference range observed intragroup might be due to differences in gender, age and quantification techniques [51,52]. However, Hb levels were normal and comparable to the PBS group. Unlike chitosan NP and their HA-coated form, LNPs used in this study sharply decreased platelet counts, which was consistent with previous observations [40,48]. Thrombocytopenia was also observed for anti-sense oligonucleotide (ASO) administered at doses above 200 mg/kg, which resulted in a halt in both the IONIS CARDIO-TTR and the NEURO-TTR phase III trials, and could be traced to the phosphorothioate (PS) backbone modification [53]. Interestingly, lymphocyte counts decreased with both lipid and chitosan-based formulations when formulated with the native immune stimulatory [39] anti-ApoB sequence.

Chitosan accumulation in the kidney did not impair kidney function, since levels of BUN and creatine remained normal. However, a drawback of our study is the lack of BUN and creatinine measurements in urine, which are more predictive than their serum counterparts, as they permit the computation of the glomerular filtration rate (GFR), which is a clinical indicator of renal function. BUN and creatinine, indirect indicators of liver health, support the absence of liver toxicity indicated by normal ALT, AST and ALP levels. Unlike uncoated and HA-coated NPs, LNPs showed a typical dose-dependent increase in transaminases, indicating transient liver toxicity [41,42,48] further accompanied by a reduction in body weight highlighting systemic (liver) toxicity. The decrease in body weight observed with LNPs [40,41,42,48] could be attributed to either the lipids [41,42,48] or the properties of the encapsulated nucleic acid payload [40]. In the present study, the decrease in body weight could be due to the general toxicity induced by the lipid system, since injections were performed with a LNA-modified sequence containing 2′Ome and phosphorotioates (PS). LPS treatment increased BUN and decreased Cr levels in serum typical of catabolic processes following the induction of cytokines in fever like symptoms or infections [54]. The decrease in BW with LPS treatment could possibly be linked with elevated cytokine levels compared with other groups that had lower (i.e., InvLNP) or no cytokine release (i.e., uncoated and HA-coated NPs). The decrease in alkaline phosphatase (with LPS detoxifying properties) following the I.V. injection of LPS could be due to malnutrition and weight loss and correlates with overt clinical signs and changes in the general appearance of mice (Table S2).

Organ and tissue toxicity is generally recognized by morphological changes, immune infiltration, apoptosis and/or necrosis. In the current study, no morphological changes, including an absence of infiltrating neutrophils, apoptotic and/or necrotic cells were observed in main organs upon single (Supplemental Figures S4–S6) and multiple injections, further confirming the safety of uncoated and HA-coated NPs. However, immune infiltration in liver was observed with high doses (8 mg/kg) of Invivofectamine^®^ 2.0 (Supplemental Figure S2) supporting immune stimulation data.

Intravenous administration caused chitosan siRNA NPs accumulation in the kidneys and promoted siRNA translocation through the glomerular basement membrane (GBM) evidenced by the intracytoplasmic localization and punctuate pattern of siRNA. The PTEC internalization of chitosan has been previously demonstrated to be dependent on the glucosamine (Glc)–megalin interaction and subsequent endocytosis [27]. HA coating modified the physicochemical properties of NPs, with a shift in size and ζ-potential indicating effective electrostatic coating, without modifying the kidney-targeted biodistribution pattern possibly via CD44 internalization. Indeed, PTECs express at least five CD44 splice variants playing an important role in HA internalization [55]. In addition, the HA-dependent colloidal stability of NPs in serum [23] could decrease in circulation due to shedding, exposing the chitosan–siRNA core (N:P 2) to accumulate in PTEC via megalin-mediated endocytosis. Independent of the observed PTEC accumulation, the mechanism of NP translocation through GBM still remains unclear, since fenestration and ECM restrict the translocation and diffusion of NPs. Alternative delivery through the fenestrated peritubular capillaries could occur but faces diffusion challenges through the negatively charged interstitium.

We next examined the efficacy of our NPs to induce target-specific knockdown. The glyceraldehyde 3-phosphate (GAPDH) gene was selected as a target due to its ubiquitous expression in tissues and the availability of in vivo validated, and chemically modified, siRNA sequences. In this study, functional GAPDH knockdown in the kidney cortex was achieved upon three injections of uncoated NPs. GAPDH enzymatic activity was reduced in the kidney lysate by around 55% and 45% using low (10 kDa) and high (120 kDa) Mn chitosan, respectively, which was confirmed by the Western blot analysis and qualitative immunohistochemistry. Unlike uncoated chitosan, HA-coated NPs accumulated in the kidney but did not induce target knockdown. This result could be explained by the need of excess chitosan (N:P 5 in uncoated vs. 2.5 in HA-coated) to promote endosomal release [56], which is possibly through the proton sponge effect. Therefore, it is likely that HA-coated NPs formulated at an N:P:C ratio of 2.5:1:2 can translocate to the cytoplasm of PTEC but remain sequestrated in endolysosomal compartments due to the poor endosomal buffering capacity and reduced proton sponge effect. In addition, the negatively charged HA molecule, if co-localizing with chitosan, could contribute to lower endosomal release by masking positive charge in the endosome, therefore reducing the capacity of endocytosed chitosan to mediate endosomal rupture. In contrast to HA-coated NPs (N:P:C ratio of 2.5:1:2), uncoated chitosan formulations prepared at an N:P ratio of 5 contain around 70% free chitosan [57] that could co-localize in PTEC endosomes and promote endosomal rupture, explaining the observed efficacy. In contrast to chitosan, Invivofectamine^®^ LNPs accumulated in liver (Supplemental Figure S2) and induced target knockdown (Supplemental Figure S7), as seen before [58]. Lower knockdown levels with LNPs in this study than seen before [58,59] could be explained by differences in target gene half-lives (t1/2).

Compared to the potency of LNPs in biopharmaceutical pipelines (~70–90%) [60,61], the lower functional target knockdown obtained with our system (~50–60%) could be explained by the half-life of the target gene, potency of the siRNA, and tissue-dependent technical challenges. Given that chitosan accumulates in PTECs (minor cell subtype of the kidney) versus LNPs in hepatocytes (predominant cell-type in the liver), an assessment of target knockdown using conventional techniques (e.g., qPCR, enzymatic activity, Western blotting) that average expression levels across all cell types in the tissue sample is inevitably underestimated. Therefore, the functional knockdown obtained in this report underestimates the true efficiency of our system to silence a target gene in PTECs, highlighting that the precise evaluation of knockdown requires the development of methods capable of estimating knockdown in a specific subset of cells composing an organ.

Taken together, our findings are critically important in revealing that uncoated and HA-coated NPs display no toxicity along with the extrahepatic delivery of siRNA leading to functional knockdown in kidney cortices. The efficacy of our uncoated system in inducing functional target knockdown in PTECs specifically differentiates it from cyclodextrin-based NPs accumulating in the glomerulus and podocytes [62]. This study also highlights the potential of the HA-coated chitosan hybrid system as a potential system that accumulates in the kidney and could be delivered at high doses without hemolytic and/or adverse events. Further investigation is needed to elucidate the mechanism of PTEC accumulation and lack of knockdown efficacy observed with the HA-coated system in this report despite similar distribution properties.

## 5. Conclusions

Uncoated chitosan NPs showed hemolytic potential in a dose and Mn-dependent manner abrogated by HA coating. Unlike lipid-based NPs and liposomes, uncoated and HA-coated chitosan NPs did not induce pro-inflammatory Type-I cytokines except KC. Toxicological profiling showed that both uncoated and HA-coated chitosan NPs injected at low and high doses were safe. LNPs (Invivofectamine^®^) induced a dose-dependent cytokine release and caused acute toxicity. In vivo biodistribution showed a cytoplasmic accumulation of siRNA in the proximal tubular epithelial cells of the kidney, with a clear role for chitosan, whether uncoated or HA-coated, in improved bioaccumulation. Uncoated chitosan nanoparticle efficacy showed 50–65% functional knockdown with clear confinement to the kidney cortex after I.V. administration. Contradicting our starting hypothesis, we found HA coating to reduce knockdown sharply despite accumulation in the kidney cortex. Taken together, our data indicate that chitosan NPs are safe delivery systems with the potential to treat kidney diseases, specifically in PTEC-related pathologies.

## Figures and Tables

**Table 1:** Characterization of chitosans tested in this study. Chitosans are denoted according to their chemical composition using the nomenclature [DDA-Target Mn] and are represented in the first column of the table. The degree of deacetylation (DDA) was determined by 1H NMR. The number and weight average molecular weight (Mn and Mw) were determined by gel permeation chromatography (GPC). The polydispersity index (PdI) was calculated as Mw/Mn. The degree of polymerization (Dp) or chain length was computed as.

Chitosan	DDA (%)	Mn (kDa)	Mw (kDa)	PdI	Dp
92-10	92.0	9.0	13.7	1.52	55
92-120	91.9	138	181	1.31	836

## Data Availability

Dataset available on request from the authors.

## Supplementary Materials

The following supporting information can be downloaded at https://www.mdpi.com/article/10.3390/polym16172547/s1, Figure S1: Hemocompatibility profiling of uncoated and HA-coated chitosan–siRNA nanoparticles via erythrocyte aggregation; Figure S2: In vivo biodistribution of Invivofectamine® 2.0 and naked siRNA; Figure S3: Cytokine levels pre-injection of Invivofectamine® 2.0, uncoated and HA-coated chitosan siRNA nanoparticles into CD1 mice; Figure S4: Histopathological comparison of liver and kidney tissue sections following intravenous administration of low doses of uncoated and HA-coated nanoparticles; Figure S5: Histopathological comparison of spleen, heart and lung tissue sections following intravenous administration of high doses of uncoated and HA-coated nanoparticles; Figure S6: S. 6 Histopathological comparison of liver and kidney tissue sections following intravenous administration of high doses of uncoated and HA-coated nanoparticles; Figure S7: In vivo target knockdown in liver using invivofectamine lipid nanoparticles; Table S1: Clinical signs collected following multiple injection of LNPs, uncoated and HA-coated chitosan-siRNA nanoparticles; Table S2: Clinical signs collected following single ascending dose of LNPs, uncoated and HA-coated chitosan–siRNA nanoparticles; Table S3: Sequence of siRNAs.
