# Supplementary material for: Chitosan siRNA Nanoparticles Produce Significant Non-Toxic Functional Gene Silencing in Kidney Cortices
Source: Polymers (Basel). 2024 Sep 9;16(17):2547. doi: 10.3390/polym16172547 (PMC11398103; doi:10.3390/polym16172547)
Supplement: Supplementary file 1 [file polymers-16-02547-s001.zip › Alameh 2024 polymers-3159194 Supp figures-R1.pdf]

Supplementary Figures

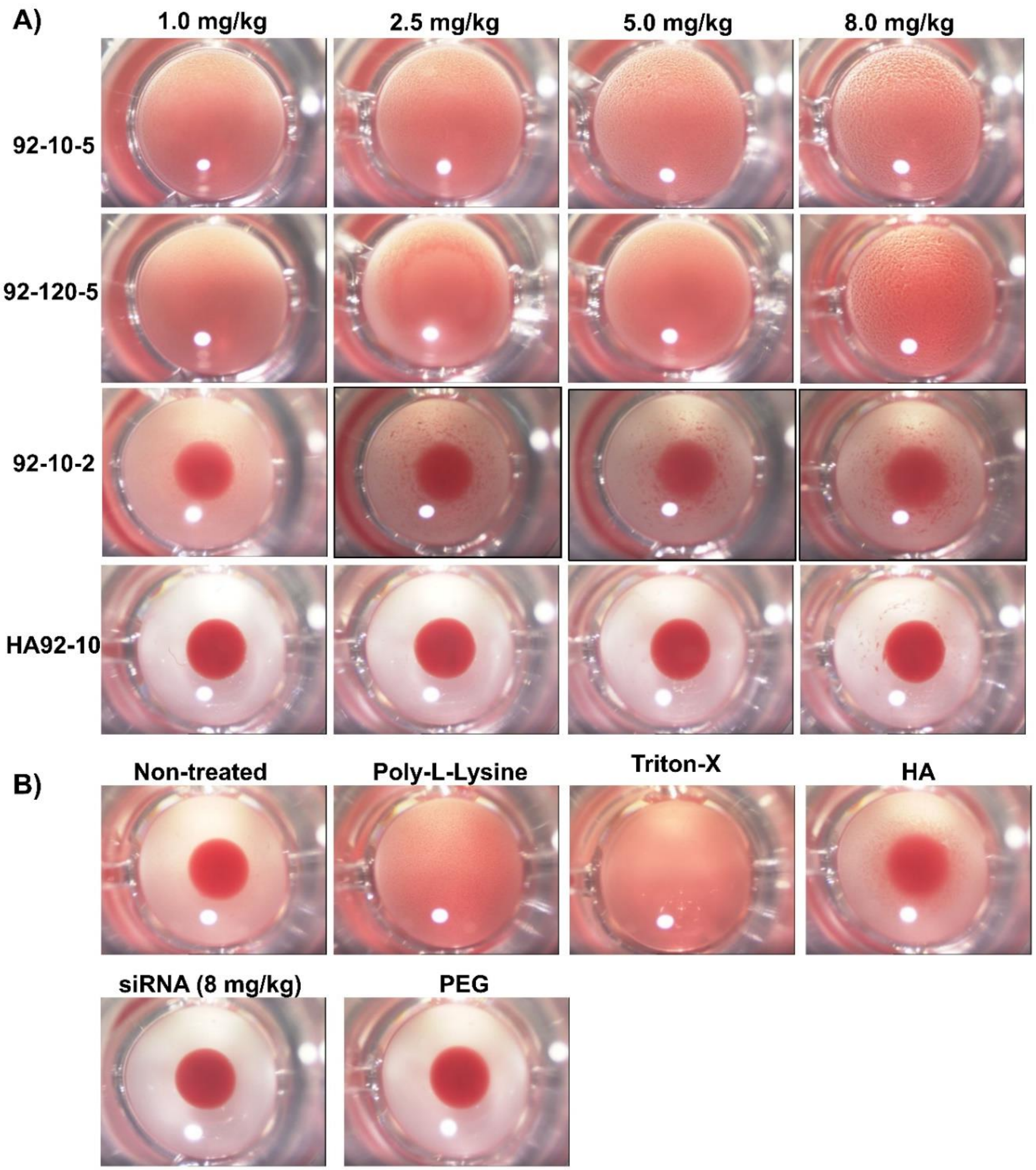
A)
1.0 mg/kg
2.5 mg/kg
5.0 mg/kg
8.0 mg/kg
92-10-5
92-120-5
92-10-2
HA92-10
B)
Non-treated
Poly-L-Lysine
Triton-X
HA
siRNA (8 mg/kg)
PEG

Figure S1. Hemocompatibility profiling of uncoated and HA coated chitosan-siRNA nanoparticles via erythrocyte aggregation. Low (10 kDa) *versus* high (120 kDa) molecular weight chitosans were formulated with HPLC-grade siRNA at an N:P ratio of 5. HA coated formulations were formulated at an N:P:C of 2:1:1.5. Increasing doses of siRNA were mixed with human pooled blood, incubated and imaged for qualitative assessment of hemagglutination. In the absence of hemagglutination, erythrocytes (RBC) deposit in the bottom of the U shaped well and form a ring (see Non-treated, PEG, siRNA in **B**). In contrast, when hemagglutination occurs, aggregates become visible, stay suspended in solution and depending on the extent of agglutination may prevent the formation of the ring. **A)** Treatment samples at different doses of siRNA. **B)** Controls.

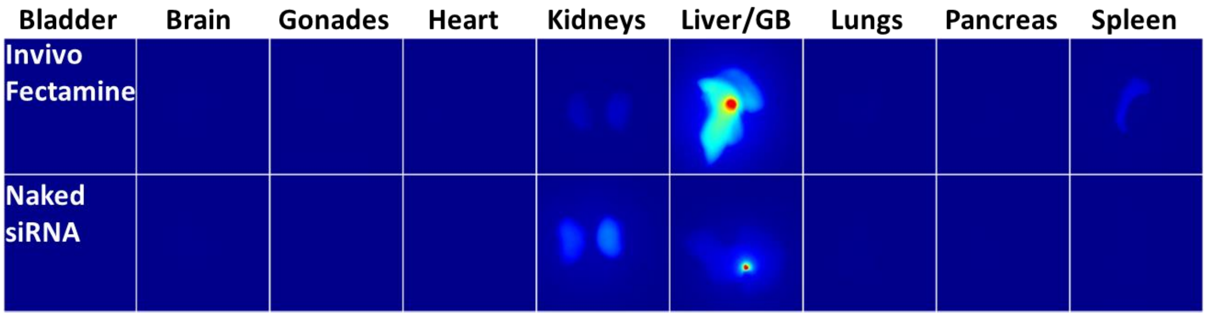

Figure S2. *In vivo* Biodistribution of Invivofectamine® 2.0 and naked siRNA. The DY^{647} fluorophore-labeled siRNA was administered at a dose of 0.5 mg/kg and organs were imaged *ex-vivo* 4 hours post-administration.

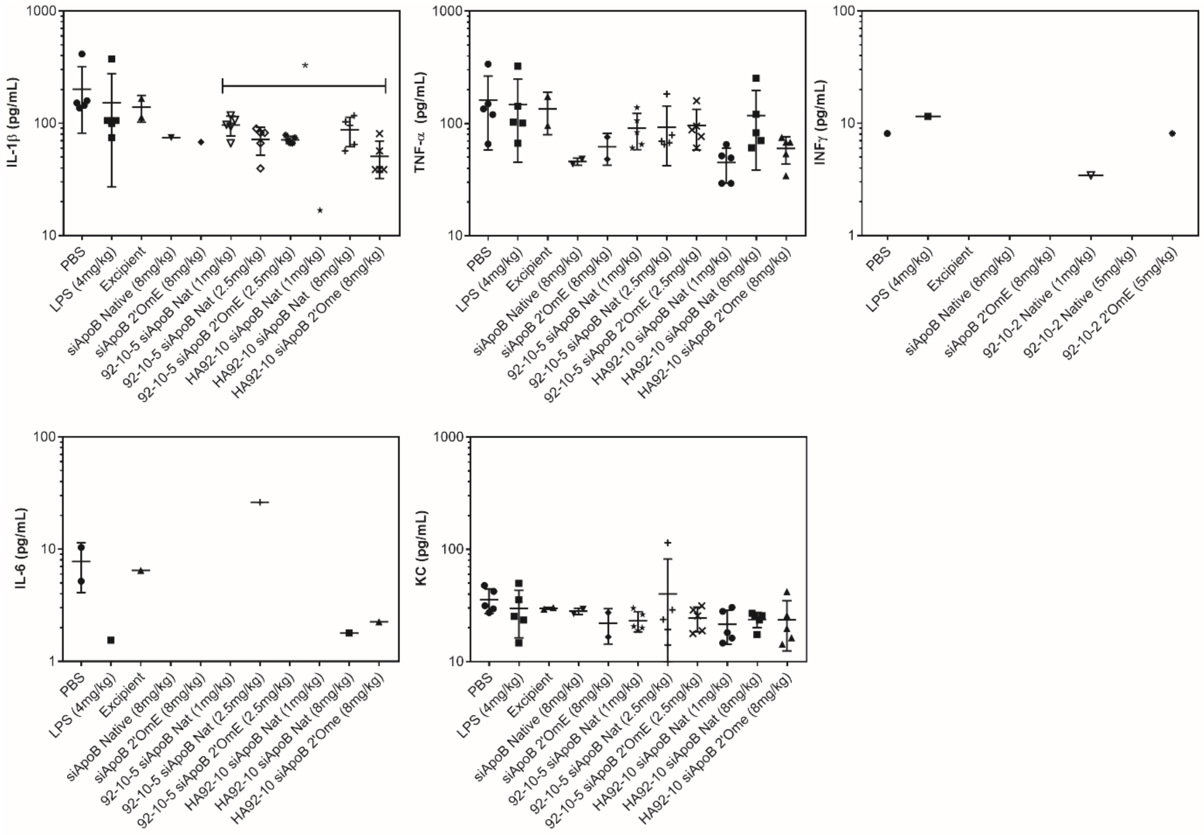

Figure S3. Cytokine levels pre-injection of Invivofectamine® 2.0, uncoated and HA-coated chitosan siRNA nanoparticles into CD1 mice. PBS (Phosphate buffered saline), LPS (Lipopolysaccharide), siApoB Nat (unmodified anti-ApoB siRNA sequence), siApoB 2'Ome (2'O methyl modified anti-ApoB siRNA sequence), and HA (Hyaluronic acid, 866kDa). Mice were intravenously injected with test articles, serum collected and analyzed 4 hours post injection using the BioPlex 200 system. Each symbol represents an animal. Note: In order to not artificially manipulate the average, cytokine levels (animals) that were below the range of detection (< OOR) were excluded and not considered as 0 or LLOQ (pg/mL).

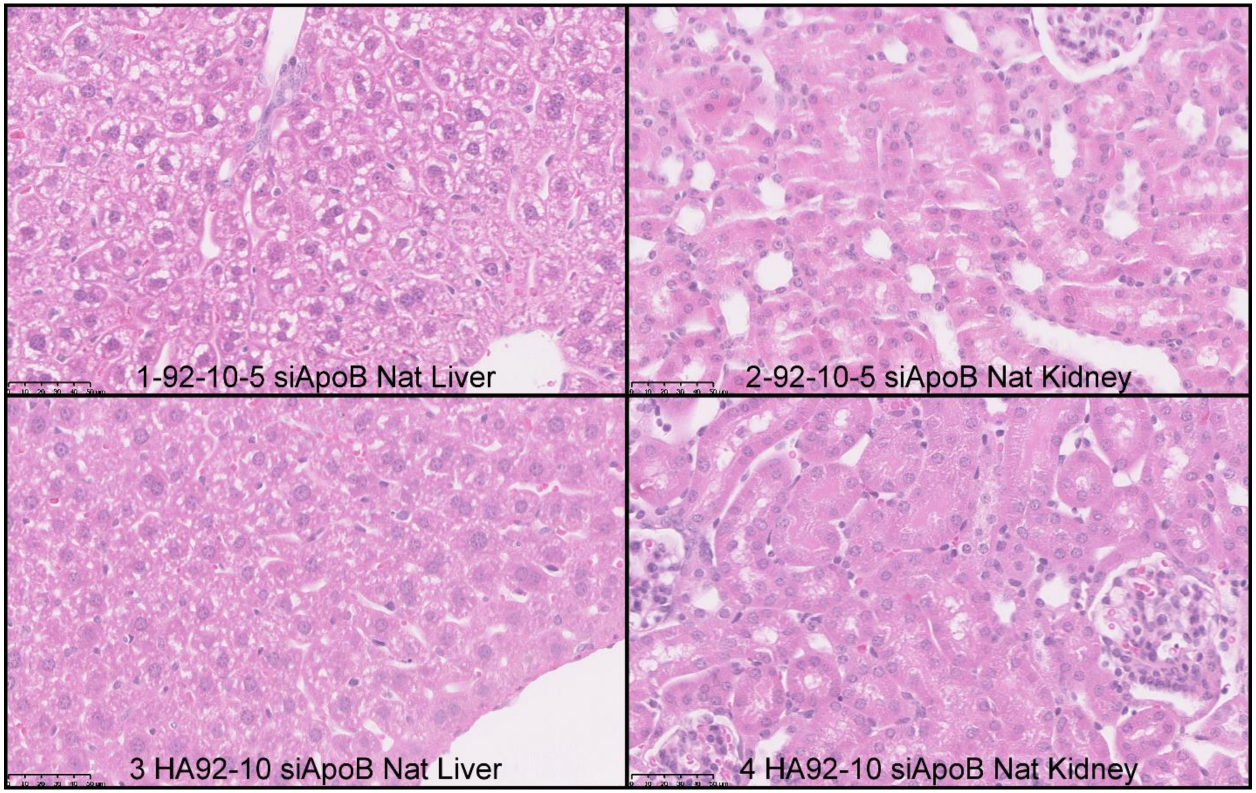

Figure S4. Histopathological comparison of liver and kidney tissue sections following intravenous administration of low doses of uncoated and HA coated nanoparticles. Uncoated and HA coated nanoparticles were formulated with unmodified (siApoB Nat) at an N:P:C ratio of 5:1:0 for uncoated and 2:1:1.5 for HA coated formulations, freeze dried, rehydrated using excipients and intravenously injected at a dose of 1 mg/kg siRNA. Animals were euthanatized 24 hours post-administration, organ collected, fixed and processed for histopathological analysis. Tissues show absence of morphological changes, alterations, clots, apoptotic/necrotic cells or infiltration of immune cells. The scale bar corresponds to 50 µm.

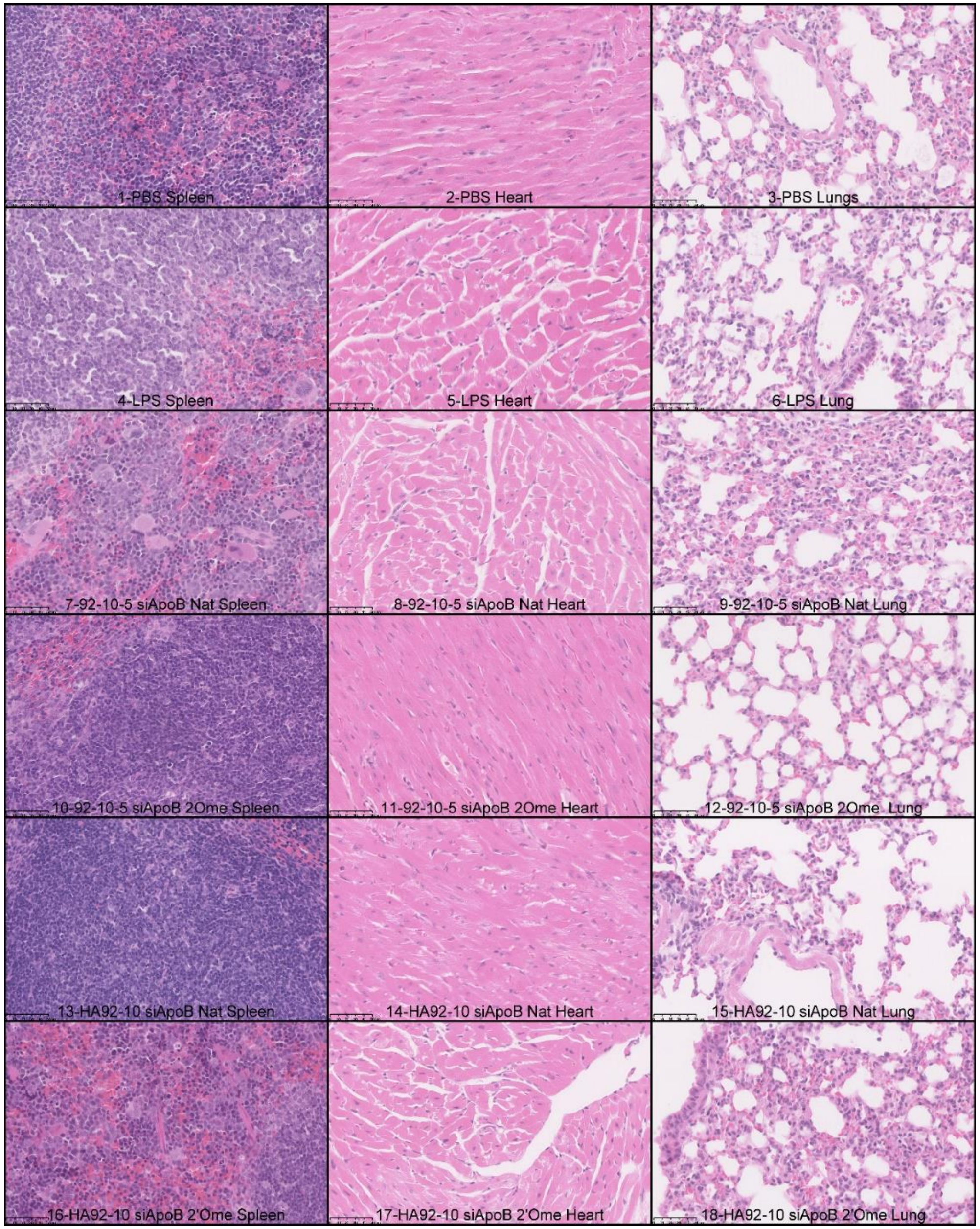
1-PBS Spleen
2-PBS Heart
3-PBS Lungs
4-LPS Spleen
5-LPS Heart
6-LPS Lung
7-92-10-5 siApoB Nat Spleen
8-92-10-5 siApoB Nat Heart
9-92-10-5 siApoB Nat Lung
10-92-10-5 siApoB 2Ome Spleen
11-92-10-5 siApoB 2Ome Heart
12-92-10-5 siApoB 2Ome Lung
13-HA92-10 siApoB Nat Spleen
14-HA92-10 siApoB Nat Heart
15-HA92-10 siApoB Nat Lung
16-HA92-10 siApoB 2'Ome Spleen
17-HA92-10 siApoB 2'Ome Heart
18-HA92-10 siApoB 2'Ome Lung

Figure S5. Histopathological comparison of spleen, heart and Lung tissue sections following intravenous administration of high doses of uncoated and HA coated nanoparticles. Uncoated and HA coated nanoparticles were formulated with either unmodified (siApoB Nat) or 2’O-methyl modified ApoB siRNA (2’Ome ApoB) at an N:P:C ratio of 5:1:0 for uncoated and 2:1:1.5 for HA coated formulations, freeze dried, rehydrated using excipients and intravenously injected at a dose of 2.5 (uncoated) and 8 (HA coated) mg/kg siRNA. Animals were euthanatized 24 hours post-administration, organ collected, fixed and processed for histopathological analysis. Phosphate buffered saline (PBS) and lipopolysaccharide (LPS) were used as controls. Organs from at least two animals per treatment group were processed and analyzed. Tissues show absence of morphological changes, alterations, clots, apoptotic/necrotic cells or infiltration of immune cells. The scale bar corresponds to 50 µm.

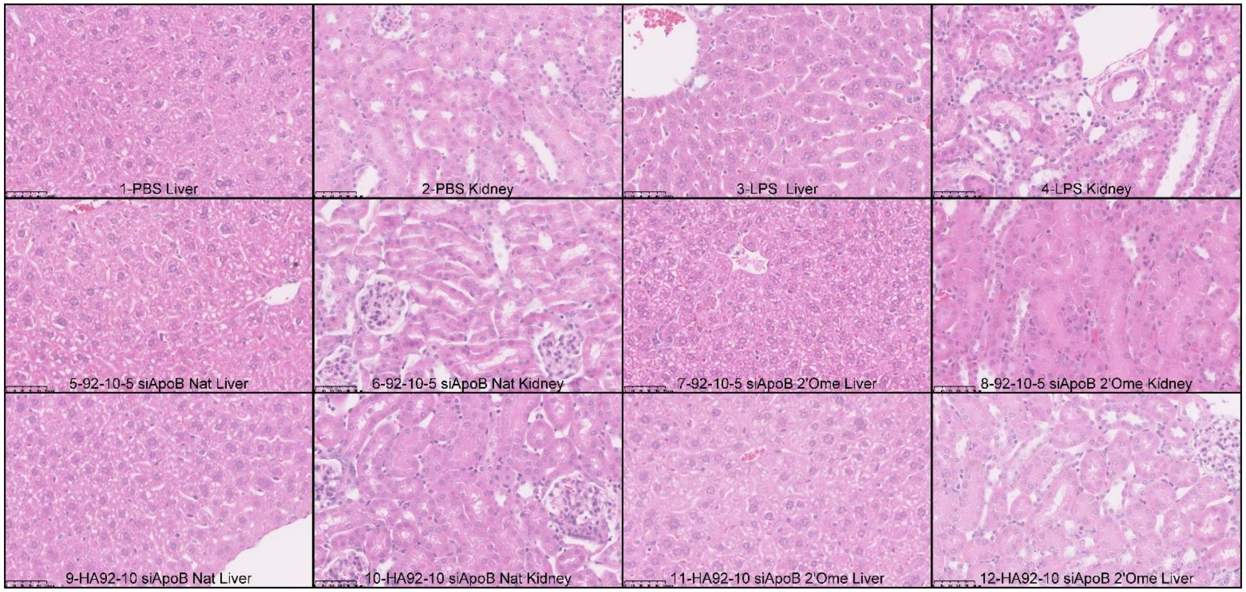

Figure S6. Histopathological comparison of liver and kidney tissue sections following intravenous administration of high doses of uncoated and HA-coated nanoparticles. Uncoated and HA-coated nanoparticles were formulated with both unmodified (siApoB Nat) and 2’O-methyl modified ApoB siRNA sequences (2’Ome siApoB) at an N:P:C of 5:1:0 for uncoated and 2:1:1.5 for HA coated formulations, freeze dried, rehydrated using excipients and I.V. injected at a dose of 2.5 (uncoated) and 8 (HA-coated) mg/kg siRNA. Animals were euthanatized 24 hours post-administration, organ collected, fixed and processed for histopathological analysis. Phosphate buffered saline (PBS) and lipopolysaccharide (LPS) were used as controls. Organs from at least two animals per treatment group were processed and analyzed. Heart, Lungs and spleen tissues form low (1 mg/kg) and high doses (2.5 and 8 mg/kg) are depicted in supplemental Figure S4. Tissues show absence of morphological changes, alterations, clots, apoptotic/necrotic cells or infiltration of immune cells. The scale bar corresponds to 50 µm.

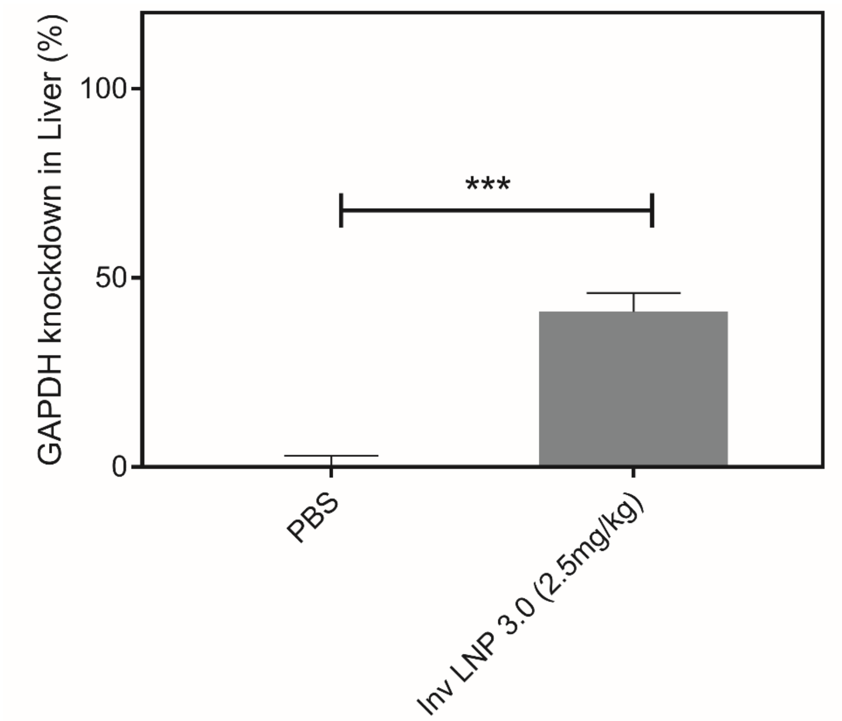

Figure S7. *In vivo* target knockdown in liver using invivofectamine lipid nanoparticles. GAPDH knockdown measured as enzymatic activity normalized per tissue mass (mg). The right liver lobes were collected, snap frozen in liquid nitogen, homogenized, protein extracted and assayed using the GAPDH KDalert™ enzymatic kit. Data represent average ± standard deviation of 5 animals/group. Statistical significance *versus* PBS-treated animals was computed with a t-test: *p< 0.01, **p <0.001, ***p < 0.00001
