# Supplementary material for: Chitosan siRNA Nanoparticles Produce Significant Non-Toxic Functional Gene Silencing in Kidney Cortices
Source: Polymers (Basel). 2024 Sep 9;16(17):2547. doi: 10.3390/polym16172547 (PMC11398103; doi:10.3390/polym16172547)
Supplement: Supplementary file 1 [file polymers-16-02547-s001.zip › Alameh 2024 polymers-3159194 Supp tables-R1.pdf]

Table S1. **Clinical signs collected following multiple injection of LNPs, uncoated and HA coated chitosan-siRNA nanoparticles. General aspect score (GAS), or the general physical aspect of the animal (i.e. hunchback position, pilo-erection, vocalization …), the natural behaviour score (NBS), or the behavioral aspect of the animal relative to its habitat and littermates (i.e. Litter aspect, activity …) and the Provoked behaviour score (PKBS), or the animal response to stimuli (i.e pen tap on the cage …) were collected by three independent scorers and reported in this table along of the frequency. ** Inv LNPs were administered once.**

TA (dose)	Clinical signs parameters	**Injections**			**Body Condition Score**	
		Injection 1 (frequency)	Injection 2 (frequency)	Injection 3 (frequency)	Before first injection	Euthanasia (8 days post injection)
PBS	GAS	0 (7/7)	0 (7/7)	0 (7/7)	3	3
	NBS	0 (7/7)	0 (7/7)	0 (7/7)	3	3
	PKBS	0 (7/7)	0 (7/7)	0 (7/7)	3	3
siGAPDH (2.5mg/kg)	GAS	0 (7/7)	0 (7/7)	0 (7/7)	3	3
	NBS	0 (7/7)	0 (7/7)	0 (7/7)	3	3
	PKBS	0 (7/7)	0 (7/7)	0 (7/7)	3	3
Inv LNP siGAPDH (2.5mg/kg)	GAS	0 (7/7)	**	**	3	3
	NBS	0 (7/7)	**	**	3	3
	PKBS	0 (7/7)	**	**	3	3
Altogen LNP (2.5mg/kg)	GAS	0 (7/7)	0 (7/7)	0 (7/7)	3	3
	NBS	0 (7/7)	0 (7/7)	0 (7/7)	3	3
	PKBS	0 (7/7)	0 (7/7)	0 (7/7)	3	3
92-10-5 siGAPDH (1mg/kg)	GAS	0 (7/7)	0 (7/7)	0 (7/7)	3	3
	NBS	0 (7/7)	0 (7/7)	0 (7/7)	3	3
	PKBS	0 (7/7)	0 (7/7)	0 (7/7)	3	3
92-120-5 siGAPDH (1 mg/kg)	GAS	0 (7/7)	0 (7/7)	0 (7/7)	3	3
	NBS	0 (7/7)	0 (7/7)	0 (7/7)	3	3
	PKBS	0 (7/7)	0 (7/7)	0 (7/7)	3	3
HA92-10 siGAPDH (8 mg/kg)	GAS	0 (7/7)	0 (7/7)	0 (7/7)	3	3
	NBS	0 (7/7)	0 (7/7)	0 (7/7)	3	3
	PKBS	0 (7/7)	0 (7/7)	0 (7/7)	3	3

Table S2. **Clinical signs collected following single ascending dose of LNPs, uncoated and HA coated chitosan-siRNA nanoparticles. General aspect score (GAS), or the general physical aspect of the animal (i.e. hunchback position, pilo-erection, vocalization …), the natural behavior score (NBS), or the behavioral aspect of the animal relative to its habitat and littermates (i.e. litter aspect, activity, nesting …) and the provoked behavior score (PKBS), or the animal response to stimuli (i.e. pen tap on the cage …) were collected by three independent scorers and reported in this table along with the frequency.**

TA (dose)	Clinical signs parameters	Time (h) post injection	
		4h (frequency)	24h (frequency)
PBS	GAS	0 (7/7)	0 (7/7)
	NBS	0 (7/7)	0 (7/7)
	PKBS	0 (7/7)	0 (7/7)
LPS (4mg/kg)	GAS	3 (7/7)	0 (7/7)
	NBS	0 (7/7)	0 (7/7)
	PKBS	3 (7/7)	0 (7/7)
siApoB Nat (8 mg/kg)	GAS	0 (7/7)	0 (7/7)
	NBS	0 (7/7)	0 (7/7)
	PKBS	0 (7/7)	0 (7/7)
siApoB 2'Ome (8mg/kg)	GAS	0 (7/7)	0 (7/7)
	NBS	0 (7/7)	0 (7/7)
	PKBS	0 (7/7)	0 (7/7)
InvLNP siApoB Nat (1 mg/kg)	GAS	0 (7/7)	0 (7/7)
	NBS	0 (7/7)	0 (7/7)
	PKBS	0 (7/7)	0 (7/7)
InvLNP siApoB Nat (8 mg/kg)	GAS	1 (7/7)	0 (7/7)
	NBS	0 (7/7)	0 (7/7)
	PKBS	1 (7/7)	0 (7/7)
InvLNP siApoB 2'Ome (8 mg/kg)	GAS	1 (7/7)	0 (7/7)
	NBS	0 (7/7)	0 (7/7)
	PKBS	1 (7/7)	0 (7/7)
92-10-5 siApoB Nat (1mg/kg)	GAS	0 (7/7)	0 (7/7)
	NBS	0 (7/7)	0 (7/7)
	PKBS	0 (7/7)	0 (7/7)
92-10-5 siApoB Nat (2.5 mg/kg)	GAS	1 (7/7)	0 (7/7)
	NBS	0 (7/7)	0 (7/7)
	PKBS	1 (7/7)	0 (7/7)
92-10-5 siApoB 2'Ome (2.5 mg/kg)	GAS	1 (4/7)	0 (7/7)
	NBS	0 (7/7)	0 (7/7)
	PKBS	1 (3/7)	0 (7/7)
HA92-10 siApoB Nat (1 mg/kg)	GAS	0 (7/7)	0 (7/7)
	NBS	0 (7/7)	0 (7/7)
	PKBS	0 (7/7)	0 (7/7)
HA92-10 siApoB Nat (8 mg/kg)	GAS	0 (7/7)	0 (7/7)
	NBS	0 (7/7)	0 (7/7)
	PKBS	0 (7/7)	0 (7/7)
HA92-10 siApoB 2'Ome (8 mg/kg)	GAS	0 (7/7)	0 (7/7)
	NBS	0 (7/7)	0 (7/7)
	PKBS	0 (7/7)	0 (7/7)

Table S3. Sequence of siRNAs

Name	Sequence
anti-ApoB siRNA sense	5'- GUC AUC ACA CUG AAU ACC AAU -3'
anti-ApoB siRNA anti-sense	5'-5' P.AUU GGU AUU CAG UGU GAU GAC AC - '3 (P denotes a phosphate group)
2'OMe modified siRNA sense	5'- GuC AuC ACA CuG AAu ACC AAu- '3
2'OMe modified siRNA anti-sense	5'-5' P.AUU GGU AUU CAG UGU GAU GAC AC - '3
anti-GAPDH sense (Locked nucleic acid modified)	5'-GGU CAU CCA UGA CAA CUU UTT-3'
anti-GAPDH anti-sense (Locked nucleic acid modified)	5'-AAA GUU GUC AUG GAU GAC-3'

Note: Lower caps are the O-Me modified nucleotides.
